# Exposure to Carbon Ions Triggers Proinflammatory Signals and Changes in Homeostasis and Epidermal Tissue Organization to a Similar Extent as Photons

**DOI:** 10.3389/fonc.2015.00294

**Published:** 2016-01-08

**Authors:** Palma Simoniello, Julia Wiedemann, Joana Zink, Eva Thoennes, Maike Stange, Paul G. Layer, Maximilian Kovacs, Maurizio Podda, Marco Durante, Claudia Fournier

**Affiliations:** ^1^Department of Biophysics, GSI Helmholtzzentrum für Schwerionenforschung, Darmstadt, Germany; ^2^Department of Biology, Technische Universität Darmstadt, Darmstadt, Germany; ^3^Department of Dermatology, Darmstadt Hospital, Darmstadt, Germany; ^4^Hochschule Darmstadt, Darmstadt, Germany

**Keywords:** human skin equivalent, keratinocytes, differentiation, apoptosis, inflammation, proliferation, ionizing irradiation, carbon ions

## Abstract

The increasing application of charged particles in radiotherapy requires a deeper understanding of early and late side effects occurring in skin, which is exposed in all radiation treatments. We measured cellular and molecular changes related to the early inflammatory response of human skin irradiated with carbon ions, in particular cell death induction and changes in differentiation and proliferation of epidermal cells during the first days after exposure. Model systems for human skin from healthy donors of different complexity, i.e., keratinocytes, coculture of skin cells, 3D skin equivalents, and skin explants, were used to investigate the alterations induced by carbon ions (spread-out Bragg peak, dose-averaged LET 100 keV/μm) in comparison to X-ray and UV-B exposure. After exposure to ionizing radiation, in none of the model systems, apoptosis/necrosis was observed. Carbon ions triggered inflammatory signaling and accelerated differentiation of keratinocytes to a similar extent as X-rays at the same doses. High doses of carbon ions were more effective than X-rays in reducing proliferation and inducing abnormal differentiation. In contrast, changes identified following low-dose exposure (≤0.5 Gy) were induced more effectively after X-ray exposure, i.e., enhanced proliferation and change in the polarity of basal cells.

## Introduction

The increasing application of charged particles in radiotherapy motivates our assessment of inflammatory reactions and homeostasis of tissue exposed to carbon ions and to compare the response to X-rays. In the current study, we focus on the analysis of cell death, proliferation, differentiation, and reorganization of different layers of the epidermis.

Charged particles display particular physical characteristics, such as high mass and electrical charge, resulting in an inverted depth dose profile compared to photons with a high relative dose deposition at the end of their trajectory. This enables a volume conform treatment of deep-seated tumors (1) as well as sparing of critical organs. In addition, when using ions heavier than protons, the exposure of cells or tissue in the “Bragg Peak” region at the end of the trajectory leads to a higher local intensity of ionizing events, and thereby clusters of DNA damage (2). As a consequence, an enhanced biological efficiency compared to photons is observed (3, 4).

New treatment approaches with carbon ions make use of these advantages by increasing the dose to the tumor to enhance the tumor control probability (1, 2). However, this also implies the delivery of a higher dose to the surrounding normal tissue, including skin (5). Skin reactions associated with carbon ion therapy for deep-seated tumors are reported to be moderate and comparable to classical photon exposure (6). However, dose escalation trials in particle therapy applying a higher dose via only 1–2 entrance channels may cause skin toxicity (5). A typical case is breast cancer proton therapy, where the target (lumpectomy cavity) is shallow, and therefore skin toxicity is the limiting factor for beam arrangement and prescription doses (7, 8).

Skin is of interest because a considerable part of the side effects occurring after radiotherapy are observed in this organ due to its sensitivity (9) and its involvement in all radiation exposures (10). Radiation effects observed in the epidermis of the skin are erythema, desquamation and, for very high doses, late necrosis. In the dermis late effects occur, i.e., persisting vascular damage and fibrosis (11–13). In addition, anti-inflammatory effects induced by low-dose radiation (14) exposure can be anticipated as they are already shown for UV exposure (15, 16).

In the work presented here, we aimed to investigate the cellular and molecular changes related to the early inflammatory response of irradiated skin, in particular the occurrence of cell death and changes in differentiation and proliferation of the epidermal cells. In this context, a comparison between X-rays and carbon ions was intended. The first experiments were performed in monolayer- and cocultures of skin cells (keratinocytes, cocultured with fibroblasts); the respective results are reported in the supplement.

Based on these results obtained in cell cultures, we used a 3D human skin equivalent (HSE) and human skin explants to approach the physiological conditions in tissue and tested the following working hypotheses:

(1) Cell death of keratinocytes does not play a major role in the inflammatory response to ionizing radiation within the first days postexposure.
(2) An early release of inflammatory cytokines in irradiated skin tissue may be elicited by other typical changes (proliferation, differentiation, and tissue organization) than cell death.
(3) Taking into account previous knowledge about irradiation-induced changes in tissue, we hypothesize that activation of proliferation, differentiation, and tissue organization may be affected.
(4) Carbon ions, delivered to normal skin under therapy conditions, induce similar effects related to inflammation, proliferation, differentiation, and tissue organization compared to the same physical doses of photons.

Throughout the assessment of cell death, cytokine release, homeostasis, and tissue organization, the effects of carbon ions, using an extended Bragg Peak as a therapy-like configuration, were compared to X-rays. As the efficiency of carbon ions in inducing the respective effects has not been reported for skin cells and tissue before, we have chosen the same low and moderate doses to compare the radiation qualities and, in addition, a high X-ray dose to take into account a potential higher but not yet determined efficiency of carbon ions. A considerable number of data sets on non-ionizing UV-B exposure are available, and therefore UV-B irradiation served as a reference, and the respective results for all model systems used are reported in the supplement.

## Materials and Methods

### Tissue Culture

Human full-thickness skin equivalent constructs (EpiDermFT™), referred to as HSE herein, were purchased from MatTek Corporation (Ashland, MA, USA) and cultured according to the manufacturer’s protocol. The HSE consists of an epidermal layer composed of normal human epidermal keratinocytes, which is not submerged in culture medium and a dermal layer built up of fibroblasts and extracellular matrix (collagen1). All HSE constructs were equilibrated for at least 16 h before the experiments were started. During irradiation, the samples were maintained in PBS (Biochrom; Berlin, Germany) and fresh medium was added after irradiation. Media exchange was repeated on a daily basis until the experiment was terminated.

Human skin tissue explants were obtained from surgical discard (Dermatology Clinic, Darmstadt, Germany). The study was approved by the Local Ethics Committee (FF136/2014). The skin was washed in PBS, cut into small pieces (5 mm × 5 mm) and explanted in cell culture inserts (BD Falcon, Heidelberg Germany). The membrane of the inserts was in contact with medium (RPMI 1640, with 10% FCS and 2% Pen/Strep; all Biochrom, Berlin, Germany). The skin explants were cultured under standard conditions.

### Irradiation

X-ray irradiation (X-RAD 320 R X, 250 kV, 16 mA) of HSE was performed with a dose rate of 1 Gy/min (0.5–10 Gy).

Carbon ion irradiation (0.5–2 Gy) was performed using a pencil beam in a spread-out Bragg peak (SOBP) with 20.0 mm width equivalent to a depth of 5 cm in water (110–145 MeV/μm; LET 100 keV/μm), at the heavy-ion synchrotron (SIS) at GSI Helmholtzzentrum für Schwerionenforschung (Darmstadt, Germany). A subset of carbon ion irradiations has been performed with the same parameters of exposure at the heavy-ion synchrotron of the Heidelberg Ion-Beam Therapy Center HIT (Heidelberg, Germany).

For carbon ion irradiation, the HSE were positioned vertically. In order to protect the samples from drying out, a sterile gaze soaked with prewarmed PBS was put in the wells under the membrane, and the wells were closed with Parafilm during exposure, which typically took 10 min.

### Histochemistry, Immunohistochemistry, Imaging, and Quantitative Analysis

For histological analyses, HSE was fixed in a 4% PFA-solution, processed for paraffin embedding, and cut into 5 μm sections using a microtome (RM2235; Leica Microsystems, Wetzlar, Germany). For hematoxylin and eosin (H&E) staining, slides were deparaffinized, rehydrated, and stained according to commonly used procedures (17).

For immunostaining, the sections were deparaffinized, rehydrated, treated with 10 mM citrate acid buffer (pH 6.0), and heated in a microwave to unmask the antigens. After rinsing in deionized water, the slides were incubated in 0.3% H_2_O_2_ for 30 min to block the endogenous peroxidase activity. After washing in PBS (three times), non-specific binding sites were blocked by incubating the sections with blocking solution (1.5% normal goat serum in PBS with 0.1% (v/v) Triton X-100). Finally, the slides were incubated with the primary antibody at 4°C overnight. Used antibodies and dilutions were: rabbit anti-active caspase-3 (Ab-2; Calbiochem, San Diego, CA, USA; 1:100), rabbit anti-Ki67 (SP6, ab16667; Abcam, Cambridge, UK; 1:100), and rabbit anti-E-Cadherin (EP700Y; ab40772; Abcam, Cambrigde, UK; 1:500). The detection of the binding of the primary antibody was performed with the Ultra-Sensitive ABC Peroxidase rabbit IgG staining kit (Thermo Scientific, Waltham, MA, USA) and the ImmPACT VIP-Peroxidase substrate kit (Vector, Burlingame, CA, USA) or SigmaFast-DAB-Tablets (Sigma, St. Louis, MO, USA) according to the manufacturer’s protocol. The nuclei were counterstained with hematoxylin and the slides were dehydrated, cleared in xylene, and mounted. HSE, submerged entirely with medium, was used as positive control for apoptosis.

Tissue sections were imaged using an Olympus BX61 microscope with an E-330 camera (Olympus, Hamburg, Germany). For the quantitative or semiquantitative analysis, 20 pictures per sample were taken with a 40-fold magnification. Pyknotic cells in the stratum corneum (parakeratosis) and in the viable epidermis were counted by eye and the mean per field of view was calculated. Ki67-positive cells (proliferation) were also counted by eye and normalized on the total number of basal cells. The thickness of the stratum corneum and the viable epidermis were measured using the software Image J. The thickness of the stratum corneum was normalized on the thickness of the viable epidermis. For the semiquantitative analysis of the structure of the basal layer, for each picture, it was evaluated if the cells in the basal layer were palisadic, in part or completely cobblestoned; the fraction of pictures displaying the respective characteristic is given. Each analysis was performed from two independent experiments, in total for four samples (*n* = 4, *N* = 2); values are given as SEM.

### Western Blot

The HSE epidermis was separated mechanically from dermis and lysed separately in RIPA buffer as previously described (18). In addition, tissue was homogenized with a pestle and with ultrasound treatment. Proteins were loaded (10 μg) and separated on 12% SDS-polyacrylamide gels, and then transferred to polyvinylidenfluoride membranes (Immobilon-P; Merck Millipore, Billerica, MA, USA). After blotting, the membranes were washed and incubated overnight at 4°C in 5% dry milk (Carl Roth GmbH, Karlsruhe, Germany) in Tris-buffered saline to reduce non-specific binding. Membranes were incubated with the primary antibodies for 2 h at room temperature.

Primary antibodies used were rabbit anti-caspase-3 (Cell Signaling, Danvers, MA, USA; 1:1000) and rabbit anti-PARP (46D11; Cell Signaling, Danvers, MA, USA; 1:1000). GAPDH (rabbit anti-GAPDH, Cell Signaling, Danvers, MA, USA; 1:1000) and α-Tubulin (mouse anti-α-Tubulin; Sigma, Steinheim, Germany; 1:4000) were used as a loading control. HaCaT cells, irradiated with 10 Gy X-rays and lysed 5 days after exposure, were used as a positive control. After washing, the membrane was incubated with a horseradish peroxidase-conjugated secondary antibody for 1 h at room temperature (anti-mouse IgG or anti-rabbit IgG HRP linked antibody; GE Healthcare, München, Germany; 1:10,000). Protein expression was visualized using enhanced chemiluminescence (Pierce ECL Plus Western; Thermo Scientific, Waltham, MA, USA) according to the manufacturer’s instructions and detected on a X-ray film.

### Enzyme-Linked Immunosorbent Assay

To quantify the levels of released cytokines in the medium of HSE, ELISA kits for the detection of TNF-α, IL-2, IL-6, IL-8, IL-10, TGF-β (all ELISA Ready-SET-go!; eBioscience, San Diego, CA, USA), and IL-1α (Platinum ELISA, eBioscience, San Diego, CA, USA) were used according to the manufacturer’s protocol. The measured values for each sample were normalized on the controls. In the first step, we checked the cytokine release from each sample before irradiation separately, but as the values were very similar to each other, this additional normalization step according to Varnum et al. (19) was not pursued. The concentration of HMGB1 in the medium of HSE was measured using an ELISA kit (human HMGB1; Cloud-Clone-Corp., Houston, TX, USA), according to the manufacturer’s instructions and normalized on the controls.

### Statistical Analysis

Unless stated otherwise, the error bars represent the mean ± SEM. Statistical significance was tested using a Student’s *t*-test. The number of independent irradiation experiments (*N*) and the total number of samples (*n*) are mentioned in the figure legends. At least two irradiation experiments and four samples were analyzed.

## Results

The results obtained in keratinocytes (normal human epidermal keratinocytes; NHEK), either in monolayers or cocultured with fibroblasts (normal human epidermal fibroblasts; NHDF), are presented in the supplement. The results obtained in a 3D HSE and in human skin explants are presented in the following paragraphs for X-ray and carbon irradiation, for UVB exposure in the supplement.

### Induction of Apoptosis

We first assessed clonogenic cell survival after radiation exposure (Figure S1 in Supplementary Material). As expected, the dose–response curve shows a typical shoulder for X-ray, whereas the curve is linear for carbon ions, indicating a higher efficiency of carbon ions compared to X-ray in terms of cell inactivation. Please note that for cell inactivation, monoenergetic carbon ions (170 keV/μm) were used, whereas all the following experiments have been performed with SOBP carbon ions (100 keV/μm), which corresponds to the conditions used in radiotherapy.

Then, we investigated if this cell inactivation is due to the induction of cell death during 144 h after exposure to ionizing radiation (X-rays and SOBP carbon ions) in a monolayer culture of keratinocytes. In addition, we repeated this experiment in cocultures of keratinocytes and fibroblasts. The results are shown in Figures S1, S2, and S4 in Supplementary Material. In spite of clearly detectable cytogenetic damage in terms of micronuclei formation for both radiation qualities, the results did not indicate an occurrence of apoptosis (no detection of annexin V positive cells, pyknotic nuclei, apoptotic bodies, activated caspase-3, and cleaved PARP), not even at high doses, and showed only low levels of necrosis (release of HMGB, High Mobility Group Box 1 protein, an established marker for necrosis (20, 21), Figure S5 in Supplementary Material).

From these results, we hypothesized that cell death of keratinocytes does not play a major role in the inflammatory response to ionizing radiation, at least not within the first days after exposure. To test this in tissue, we moved on using a model system of higher complexity, i.e., a commercially available, three-dimensional HSE, and for selected experiments also human skin explants. We used the same physical doses of photons and carbon ions (0.5 and 2 Gy), and in addition a higher dose (10 Gy) of photon irradiation.

**Figure 1:**
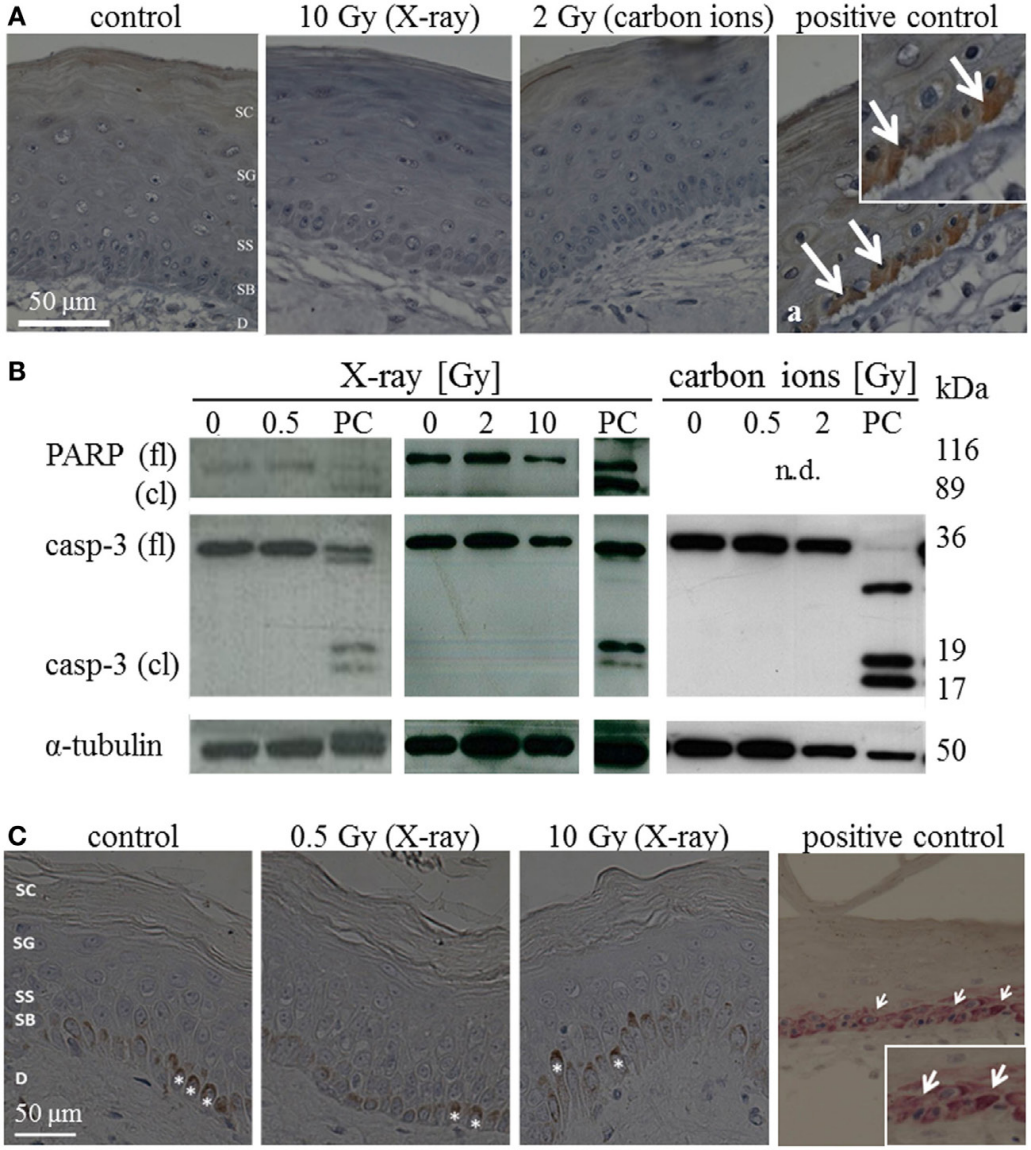
**Detection of apoptosis *in situ* and in western blot in HSE and human skin explants 24 h after irradiation with high doses of X-ray and carbon ions**. **(A)** Immunodetection of active caspase-3 (brown) in HSE; cleaved caspase-3 was not detected in the epidermis after irradiation, but in the PCs (arrows in a); *N* = 3, *n* = 5. **(B)** Western blot analysis of caspase-3 and PARP in HSE; apoptosis is detected by the presence of caspase-3 and PARP cleavage fragments (17 and 19 kDa; 89 kDa) in PCs; active/cleaved caspase-3 and cleaved PARP were not detected in the epidermis of HSE after irradiation; *N* = 2, *n* = 4. **(C)** Immunodetection of cleaved caspase-3 (pink) in human skin explants; cleaved caspase-3 was not found after low or high doses of X-rays; *: melanocytes in the basal layer (brown); *N* = 3, *n* = 5.

The occurrence of apoptosis was assessed in irradiated HSE and human skin up to 72 h after exposure. Figure 1A shows representative pictures of the immunodetection of active caspase-3 in HSE tissue sections, 24 h after exposure to moderate/high doses. In the positive control, apoptotic cells were identified in the basal layer by positive caspase-3 staining and condensed pyknotic nuclei. In contrast, no pyknotic nuclei or cells positive for active caspase-3 could be detected after irradiation, regardless of radiation quality and dose. This also applies for 72 h after irradiation (Figure S6A in Supplementary Material). Consistently, no cleaved caspase-3 and PARP (only assessed for X-ray exposure) were detected in lysates of irradiated HSE (western blot analysis for 24 h after exposure, Figure 1B, additional time points shown in Figure S6B in Supplementary Material). For X-ray exposure, these observations were confirmed in sections of *ex vivo* irradiated human skin explants where no active caspase-3 and no pyknotic nuclei were observed (Figure 1C). The use of TUNEL assay turned out to be inappropriate to detect apoptotic cells because differentiating keratinocytes showed intensive staining, irrespective of radiation exposure, and can therefore not be distinguished from apoptotic cells (not shown), which is in line with independent observations (22).

In addition, signs of necrosis were not observed morphologically and release of HMGB1 was not detectable after irradiation neither in the medium of HSE nor in the medium of human skin explants (not shown).

All in all, our results indicate no major role for early apoptosis and necrosis neither for photon nor for carbon ion exposure within 72 h after irradiation. This we conclude from the absence of caspase-3-dependent apoptosis, HMGB1 release, and typical morphological alterations, observed in a 3D HSE, and confirmed in human skin explants, irradiated *ex vivo*.

### Release of Cytokines Related to Inflammation

By analyzing cytokine release after irradiation of NHEK, an upregulation of proinflammatory cytokines on the level of gene and protein expression has been shown (12). In good agreement with published data, our own results have shown an enhanced release of IL-1α, IL-2, and TGF-β (Figure S7A in Supplementary Material, only assessed for X-rays). For IL-6 and IL-8, the measured cytokine concentration in the supernatant of the controls was below the detection limit. Thus, the relative radiation-induced increase could not be calculated reliably. The induction of IL-6 was induced by moderate doses of X-ray and UV-B irradiation, whereas IL-8 was only inducible by a high UV-B intensity (40 mJ/cm^2^, not shown).

Of note, when the keratinocytes have been cocultured with normal dermal fibroblasts, a significant influence on the pattern of release, i.e. an inhibitory feedback loop between release of IL-1α and TGF-β, has been observed in the non-irradiated cells, which is in agreement with published data (Figure S7B in Supplementary Material) (23–26). Despite this modulating effect, a radiation-induced moderate increase in the release of IL-1α, and a clear increment of IL-6 and IL-8 release, has been detected 24 h after photon irradiation, whereas no significant change of TGF-β has been measured (Figure S7C in Supplementary Material, only assessed for X-ray).

**Figure 2:**
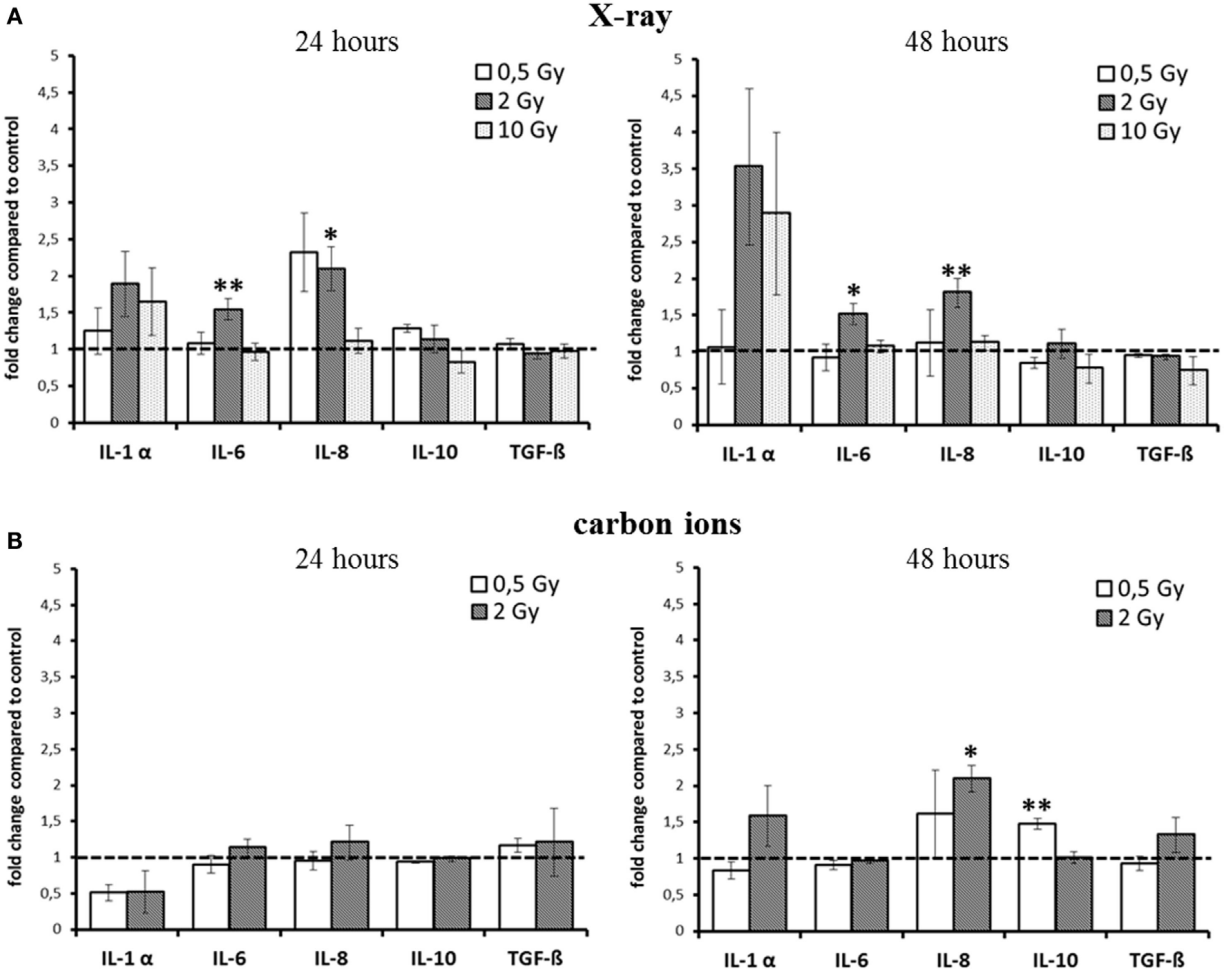
**Detection of cytokine release from HSE after irradiation with X-ray and carbon ions**. **(A)** Enhancement of IL-6 and IL-8 release 24 and 48 h after exposure to moderate doses of X-rays. **(B)** No changes 24 h after carbon ion irradiation, enhanced release of IL-10 and IL-8 was detected 48 h after irradiation with carbon ions; SEM; *p ≤ 0.05, **p ≤ 0.01; *N* = 2–3, *n* = 3–10; IL-2 and TNF-α were not detectable.

Based on these findings, we assessed the cytokine release for relevant candidates after exposure to X-rays and carbon ions in the HSE. The results are summarized in Figure 2. As the kinetics of cytokine release turned out to be different for low versus high doses and for X-ray versus carbon ion exposure, the intended comparison of the respective impact of both radiation qualities was difficult. Furthermore, the release of TNF-α und IL-2 was very low, below the detection limit in all HSE experiments, except in positive controls of human skin, which were generated by submerging the skin explant with liquid. When these human skin explants were irradiated additionally, an increase of TNF-α could be measured (not shown).

For X-ray exposure (Figure 2A), we observed a trend for an enhancement of IL-1α release 24 h after 2 and 10 Gy, whereas after 48 h, the increment was around threefold compared to the level of controls, although not significant. For the highest X-ray dose (10 Gy), the increase was the same as for 2 Gy, and for 0.5 Gy, the release was unchanged at both time points. However, this was the only change observed after exposure to 10 Gy X-rays. The release of IL-6 after X-ray irradiation was only slightly, albeit significantly enhanced for 2 Gy at both time points (1.5-fold).

The level of the chemoattractant IL-8 protein showed a more than twofold enhancement after exposure to 0.5 Gy as early as 24  h after irradiation, and the increment for 2 Gy was significant at 24 h and also at 48 h postirradiation. As in the case of IL-6, no change in IL-8 release was observed for 10 Gy at none of the time points assessed.

Notably, for carbon ions (Figure 2B), after 24 h, no increment in the release of any of the measured cytokines was observed. At 48 h after exposure, there was a trend for an enhancement of IL-1α, but not for IL-6 release. The release of IL-8 was significantly increased about a factor of two.

The cytokines that are considered to have an anti-­inflammatory effect at early times after irradiation, TGF-β and IL-10 (12, 27, 28), were not enhanced up to 48 h after exposure, regardless of the radiation quality. Although for IL-10, a significant enhancement was measured 48 h after carbon ion exposure (Figure 2B), the increment was around 1.5-fold compared to controls, raising the question about the biological significance of this modification.

Taken together, for the inflammatory cytokines IL-6 and IL-8, an enhancement within 48 h was detected after X-ray irradiation (Figure 2A). However, the observed changes were not strictly dose-dependent, and after carbon ion exposure (Figure 2B), only small changes were measured compared to the same physical doses of X-rays (0.5 and 2 Gy).

### Abnormal and Accelerated Differentiation

Epidermal homeostasis is maintained by a balance between proliferating and differentiating keratinocytes. For epithelial and other tissues (29, 30), early radiation-induced changes in proliferation, differentiation of keratinocytes, and reorganization of the epidermal layer are discussed to play a crucial role in early inflammation of the skin (31). Keratinocytes are organized in stratified layers. The basal layer (stratum basale) contains proliferating keratinocytes, which migrate into the upper layers (stratum spinosum and stratum granulosum) during their differentiation process by disconnecting from the basal membrane. During this process, morphology and protein expression profiles change. When keratinocytes finally reach the outer layer of the epidermis (stratum corneum), they have lost their nuclei and are terminally differentiated to cornified cells, which constitute the mechanical barrier of the skin, protecting the organisms against any type of external stress (10).

An abnormal pattern of morphology and differentiation is the occurrence of keratinocytes with pyknotic nuclei. If they are observed in the stratum corneum, which in healthy tissue consists of denucleated keratinocytes, this phenomenon is called parakeratosis and is associated with skin diseases (32, 33). These cells are also found in the viable part of the epidermis and in this case they are termed “sunburn cells”, as they were first described after UV exposure (34, 35).

**Figure 3:**
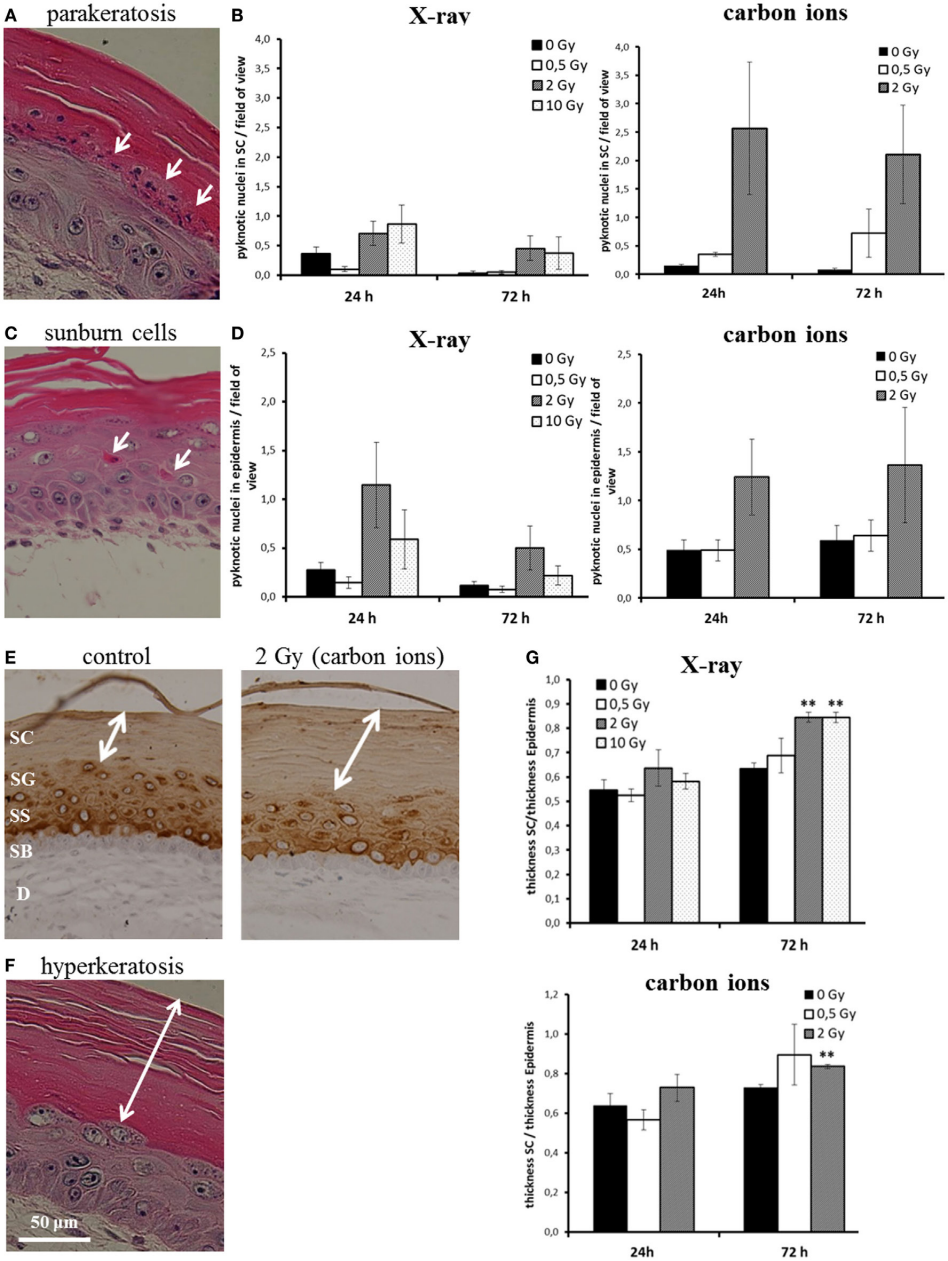
**Abnormal and accelerated differentiation in HSE after irradiation with X-ray and carbon ions**. **(A)** Pyknotic keratinocytes are observed in the stratum corneum (parakeratosis). **(B)** Quantification of parakeratosis shows a slight increase after X-ray and a more pronounced increase after carbon ion exposure. **(C)** Morphology of typical “sunburn cells“ characterized by pyknotic nuclei and an eosinophilic cytoplasm. **(D)** Quantification of “sunburn cells” shows a clear increase after 2 Gy of X-ray and carbon ions exposure. **(E)** Cytokeratin 10 expression (only in differentiating layers) in HSE 72 h after irradiation with carbon ions shows an enhanced thickness of the stratum corneum, where Cytokeratin 10 is not expressed. **(F)** Thickening of the stratum corneum (hyperkeratosis). **(G)** Quantification of hyperkeratosis shows an increase of the thickness of the stratum corneum 72 h after X-ray and carbon ion irradiation; SEM; *p ≤ 0.05, **p ≤ 0.01; *N* = 2, *n* = 4.

We assessed parakeratosis and “sunburn cells” in irradiated HSE at 24 and 72 h after exposure (Figure 3). In Figure 3A, a representative picture of parakeratosis is shown. Quantification was achieved by counting the number of pyknotic cells in the stratum corneum per field of view. As can be seen in Figure 3B, we observed parakeratosis at a low level in non-irradiated HSE (0.1–0.6 pyknotic nuclei in the stratum corneum per field of view) and an indication for an increase, albeit not statistically significant in HSE after carbon ion exposure. In Figure 3C, so-called “sunburn cells” are shown, which are not only characterized by pyknotic nuclei but also by an eosinophilic cytoplasm and the occurrence in the viable epidermis (34, 35). Quantification (Figure 3D) of these cells in the viable epidermis revealed a comparable increase 24 h after exposure to a moderate dose of X-ray and carbon ions (2 Gy), which was still persisting 72 h after irradiation. Notably, the increment was not observed for a low (0.5 Gy) and a high X-ray dose (10 Gy).

In some studies, sunburn cells are reported to be apoptotic, because the morphological alteration overlaps for part of the cells with positive staining for activated, cleaved caspase-3 (36). This was clearly not the case for the HSE in our study; in none of the experimental conditions, a colocalization of sunburn cells and caspase-3 positive staining was detected (see Figure 1; Figure S6 in Supplementary Material).

Another physiological change reported after UV-B exposure (37) is the thickening of the stratum corneum. The stratum corneum is the epidermal layer where the differentiation is terminal and Cytokeratin 10 is not expressed (38) (example shown in Figure 3E). The thickening of the stratum corneum corresponds to an accelerated differentiation leading to an accumulation of cornified cells and is considered as a protective mechanism (39). In Figure 3F, a thickened stratum corneum (so-called “hyperkeratosis”) of an irradiated HSE is depicted. For quantification, we measured the thickness of the stratum corneum and normalized this value to the thickness of the viable epidermis. As shown in Figure 3G, an increase of the stratum corneum was observed 72 h after exposure. The enhancement was significant for 2 and 10 Gy X-rays and 2 Gy carbon ions, whereas irradiation with a low dose (0.5 Gy) did not yield an effect.

The results show that abnormal differentiation patterns occur for moderate doses and were more pronounced for carbon ion than for X-ray exposure, whereas accelerated differentiation is significantly enhanced for X-ray exposure, also for a high dose, and for carbon ions, only a trend is observed. Both abnormal and accelerated differentiation is not detectable for low doses.

### Proliferation

Enhanced proliferation due to a chronically activated state of keratinocytes has been reported for human skin, where skin biopsies have been taken from patients who had undergone radiotherapy and investigated months later (31). As we have observed accelerated differentiation for moderate and high doses, we set out to investigate a potential association with enhanced proliferation at early times after irradiation.

**Figure 4:**
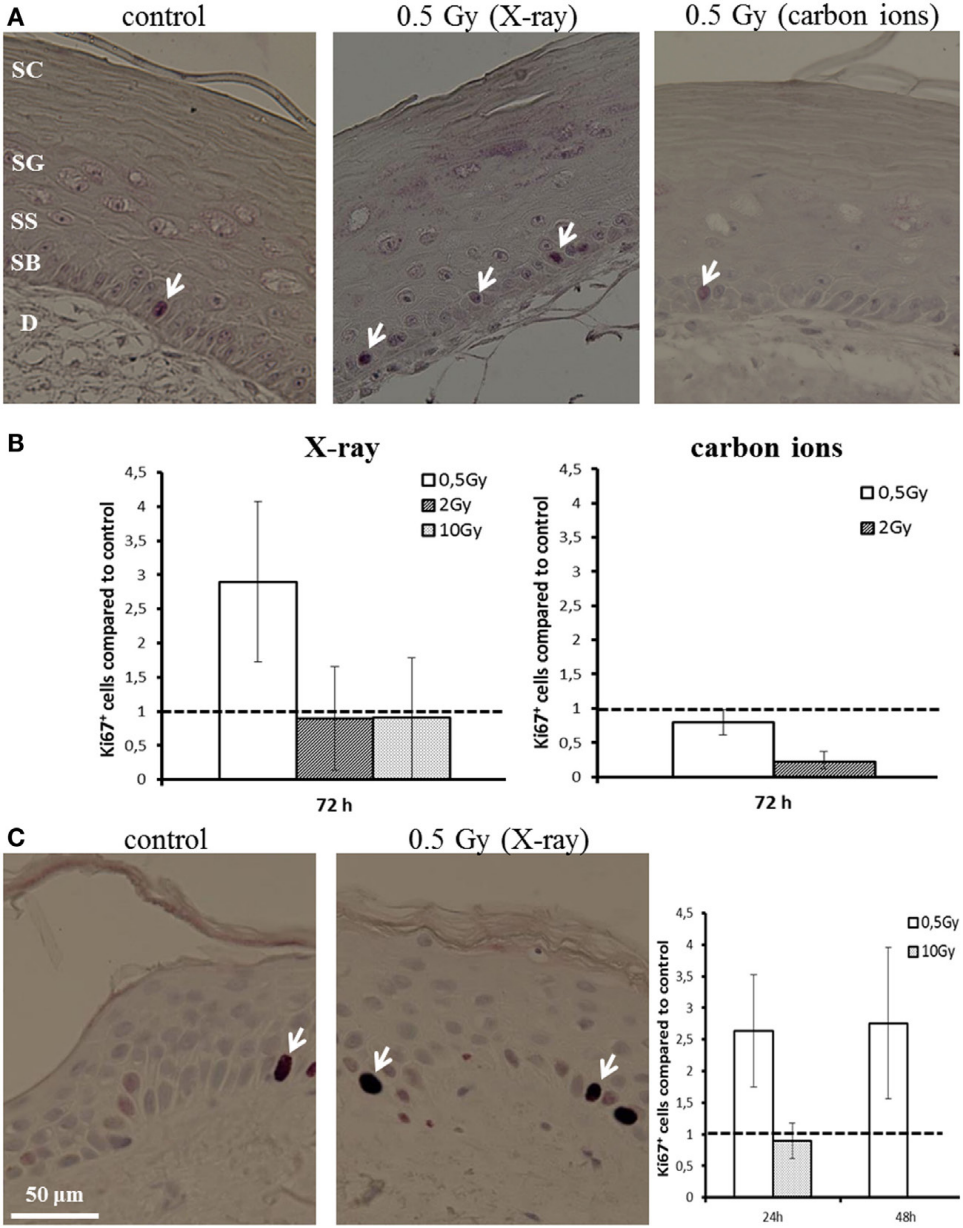
**Proliferation activity measured by Ki67 staining in HSE and human skin explants after irradiation with X-ray and carbon ions**. **(A)** Ki67-positive cells (arrows) in the basal layer of HSE. **(B)** Number of Ki67-positive cells in the HSE, normalized on the total number of cells in the basal layer and shown relative to the controls, shows enhanced proliferation after 0.5 Gy X-ray irradiation. **(C)** Ki67-positive cells in human skin explants (arrows); quantification shows an increase of proliferation after low dose of X-rays; *N* = 2, *n* = 4.

Proliferation activity was measured by Ki67 staining 72 h after irradiation of HSE. Figure 4A shows Ki67-positive cells in the basal layer. In controls, around 5% of the basal cells were positive for Ki67. Quantification of the fraction of Ki67-positive cells is depicted in Figure 4B, normalized on the level of non-irradiated HSE. An enhanced proliferation activity was observed after irradiation with a low X-ray dose (0.5 Gy), though not significant due to interexperimental variation. For higher X-ray doses and a low dose of carbon ions (0.5 Gy), no changes were observed, whereas following exposure to 2 Gy carbon ions a reduced fraction of proliferating cells was detected.

An increase in proliferation activity of the basal cells for 0.5 Gy and an unchanged activity for 10 Gy was confirmed in first experiments using explants of human skin (Figure 4C), which were *ex vivo* exposed to X-ray irradiation (24 and 48 h).

These results show an enhanced proliferation occurring only after exposure to a low dose of X-rays, but not for carbon ions, pointing to a specific effect, which is inversely correlated to increasing dose and ionizing density. According to this, at higher doses, no changes or even a reduced proliferation activity have been detected, the latter indicating an inhibition of cell cycle progression. This is consistent with the results obtained in NHEK (Figure S8 in Supplementary Material).

### Changed Polarity of the Basal Cells

The polarity of the basal keratinocytes is a prerequisite for a balanced homeostasis of the epidermal layer (40). The typical palisade-like morphology of the basal cells allows for an attachment to the basal membrane and for a regular alignment, determining the polarity of the basal cells. When the basal cells are not attached to the basal membrane, the order and structure of the basal layer is disturbed, potentially leading to uncontrolled proliferation and migration (41).

**Figure 5:**
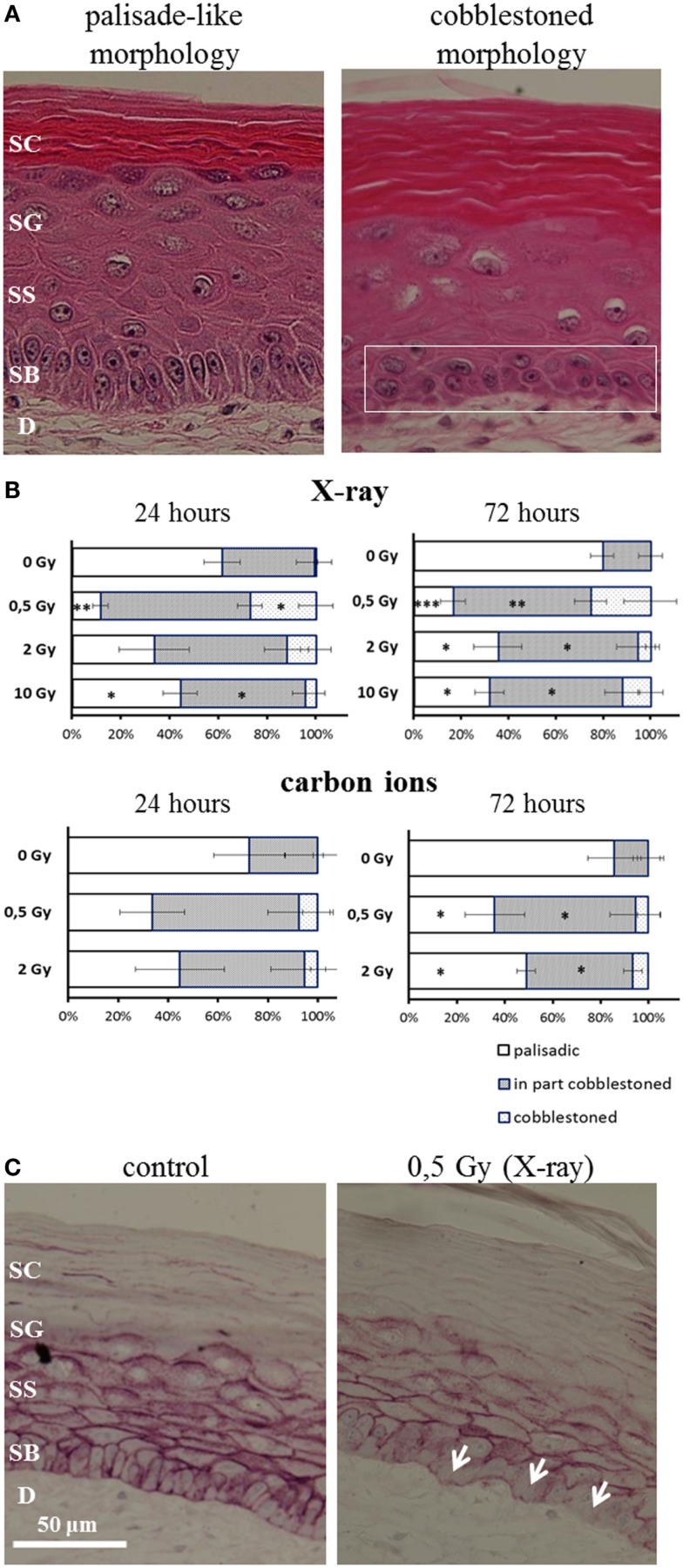
**Changed polarity of basal cells in HSE after irradiation with X-ray and carbon ions**. **(A)** Transition of palisade-like morphology of basal cells to a cobblestoned morphology indicating a change in polarity and disorganization of the basal layer. **(B)** Quantification of palisade-like morphology and cobblestoned (partial or total) morphology shows a transition for all doses of X-ray and carbon ions; most pronounced and highly significant for 0.5 Gy. **(C)** E-Cadherin staining shows a delocalization of the protein in the cells from the basal layer (arrows) 72 h after irradiation with 0.5 Gy X-rays; SEM; *p ≤ 0.05, **p ≤ 0.01; ***p ≤ 0.001; *N* = 2, *n* = 4.

After irradiation, we observed a transition from the typical palisade-like morphology to a cobblestoned morphology of the basal cells, as shown in a representative picture in Figure 5A. As quantification is difficult, we performed a semiquantitative scoring by determining if in the field of view all basal cells display a palisade-like morphology or if the cells have undergone a partial or a complete transition to a cobblestoned morphology. The semiquantitative evaluation in Figure 5B shows a shift to a cobblestoned morphology for X-ray exposure compared to controls. A transition to more areas with cobblestoned morphology was observed 24 and 72 h after irradiation, and in some fields of view, all basal cells displayed a cobblestoned morphology. Interestingly, the effect was inversely correlated with increasing dose and most pronounced after 0.5 Gy. Similar changes were found after carbon ion irradiation (Figure 5B) but less pronounced comparing the low dose (0.5 Gy) for both radiation qualities. In addition, we observed an alteration, which may be related to the described changes in morphology and polarity of basal keratinocytes, i.e., a delocalization of E-Cadherin from the cytoplasmic membrane to the cytoplasm (Figure 5C).

In summary, the transition of basal cells from a palisade-like to a cobblestoned morphology, indicating a change in polarity and disorganization of the basal layer, occurs for low and high doses, and for all radiation qualities. However, the effect is clearly more pronounced for low compared to high doses and for X-rays compared to carbon ions comparing the same physical doses.

## Discussion

The early and late skin response to ionizing radiation in classical photon therapy is clinically well known (31, 42) and constitutes a dose-limiting factor (43, 44). However, for reactions occurring within the first days in the epidermal layer of the skin, the cellular and molecular basis is explored much more intensive for UV exposure than for ionizing irradiation. For carbon ion exposure, the early radiation response of skin tissue has been investigated for the first time on a cellular level in our current study.

### Cell Death Does Not Play a Major Role in the Early Radiation Response of Skin

The onset of an inflammatory reaction is one of the first events after irradiation of skin (45), and cell death can trigger this response (14, 46). Given the well-known enhanced efficiency for cell inactivation and higher relative biological effectiveness (RBE) of two to five of carbon ions (depending on the energy) compared to photons in mammalian cell types (47–49), a careful investigation of cell death induction in epidermal cells within the first days after exposure was conducted. As expected, clonogenic survival of NHEK was reduced after X-ray exposure and even more pronounced after high LET carbon ion irradiation (170 keV/μm, Figure S1A in Supplementary Material). However, cell death was not detectable in mono and coculture of NHEK (Figures S1 and S4 in Supplementary Material, assessed for X-rays), which is consistent with reported results, where no or only a minor early increment in apoptotic cells was observed in primary keratinocytes exposed to moderate and high doses of γ-rays (50, 51). This indicates different mechanisms of clonogenic inactivation, such as accelerated differentiation, as shown for other primary cells (49, 52).

Using the more complex skin models, HSE and human skin explants, we confirmed that caspase-3-dependent apoptosis and necrosis do not play a role within the first days after radiation exposure to both X-rays and carbon ions in the assessed dose range (Figure 1). A low level of apoptosis, remaining unchanged after irradiation of the same HSE as used in our experiments, was also mentioned in an independent study (53). In biopsies of radiotherapy patients, the low basic level of apoptosis was increased only after more than 6 weeks (42), and in animal experiments, caspase-3-dependent apoptosis (22) and epidermal cell loss (54) were shown for very high doses. We conclude that apoptosis occurs only for very high doses and/or later than a few days. Early after exposure to low and moderate doses, apoptosis and necrosis do not contribute to inflammatory reactions.

### Carbon Ions and X-Rays Trigger an Early Release of ProInflammatory Signals in Irradiated HSE with Similar Efficiency

For X-ray exposure, an early upregulation of inflammatory pathways on the transcriptional level in the irradiated epidermis is well established and has been investigated in skin biopsies of radiotherapy patients, in HSE (12, 53, 55, 56), and in keratinocytes in animal and cell culture studies (50, 57). We could show in a HSE that both photon and carbon ion irradiation induce an early, significantly increased release of cytokines, which are known to trigger inflammation, such as IL-6 and IL-8, and a trend in increase of IL-1α. Anti-inflammatory cytokines (TGF-β and IL-10) were not elevated after exposure, except for a small enhancement of IL-10 at 48 h following carbon ion irradiation. This argues against an anti-inflammatory response at low doses elicited in the model systems investigated here. However, TGF-β mRNA was reported to be upregulated for high γ-ray doses (58), probably related to its key role in the late fibrotic response of skin.

In our study, comparing the same physical doses, the response to carbon ion irradiation compared to X-ray exposure was weak, detectable only after 48 h and significant only for IL-8 release (Figure 2). This indicates a similar enhancement in the release of proinflammatory cytokines after X-ray and carbon ion exposure. However, this is more a relative statement concerning the efficiency of carbon ions compared to X-rays than a result which fully represents the inflammatory response in a skin model such as a HSE, because a partially, but not fully overlapping pattern of X-ray induced cytokine release was detected in a study conducted by an independent group in the same HSE (19).

### The Differentiation of Epidermal Cells After Irradiation is in Part Abnormal and Accelerated

Typical features that might contribute to the onset of an inflammatory reaction in skin are changes in proliferation and differentiation of keratinocytes, as reported for radiotherapy patients and irradiated animals (31, 59, 60). The normal differentiation and migration process implies nuclear disintegration of the keratinocytes that have reached the stratum corneum (10). When nucleated cells are found in the stratum corneum, the differentiation process is abnormal and called “parakeratosis”. We observed parakeratosis after exposure to carbon ions (2 Gy), whereas only a weak induction was detected after irradiation with moderate and high X-ray doses (Figure 3B). In line with a change occurring at higher ionizing densities, parakeratosis was reported also for proton irradiation in an epidermis equivalent (61).

Another indicator of abnormal development is the occurrence of cells with pyknotic nuclei and eosinophilic cytoplasm, which are located in the viable epidermis. We found an increased number of those cells, albeit at a low level, after exposure to moderate doses of X-rays and, longer persisting, for carbon ions (2 Gy; Figure 3D). However, unlike “sunburn cells”, which have been observed after UV exposure (34, 35), these cells did not show positive staining for activated caspase-3 and were not found in the basal layer. This result indicates that cells with a sunburn-like morphology detected after X-ray and carbon ion irradiation can be ascribed to abnormal differentiation and that this process is not necessarily associated with classical caspase-3-dependent apoptosis. Based on the morphological similarity, the occurrence of these cells might be a prestep for parakeratosis.

In contrast to the aberrant features (parakeratosis, “sunburn cells”), which occur to a higher extent after carbon ion exposure, we found indications for non-aberrant, but accelerated differentiation after exposure to moderate and high but not for a low X-ray dose. Quantitative analysis revealed a significant enhancement of thickness of the stratum corneum (hyperkeratosis) for X-rays and for carbon ions. Similar observations in an epidermal skin equivalent are reported for proton exposure (61) and less pronounced for higher LET ions (62). All in all, our own and published data indicate for X-ray and charged particles of the lower LET range that the induced imbalance of the differentiation process manifests as accelerated and not really aberrant as observed for higher LET radiation qualities.

### The Proliferation Activity of Basal Cells is Enhanced for a Low Dose of X-Rays

Differentiation and cell proliferation are directly associated; therefore, we also studied the proliferation activity of the basal cells in the HSE, which we found to be enhanced for a low X-ray dose (Figure 4). Notably, in human *ex vivo* irradiated skin, we could confirm the enhanced proliferation activity of epidermal cells induced by low X-ray doses (Figure 4C). For higher X-ray doses and for carbon ions, the proliferation activity was unchanged or even inhibited, which is in line with results from animal photon studies (22, 54, 60, 63) and consistent with the cell cycle arrest that we observed in NHEK (Figures 4A,B; Figure S8 in Supplementary Material).

Our results suggest that increased proliferation is a low-dose effect, which is induced within a few days after exposure. Furthermore, the effect seems to be related to ionizing density, which is endorsed by the observation of an increased proliferation after exposure to charged particles with a relatively low LET [protons (61) and oxygen (62)], which was not detected for heavy ions with a higher LET in the HSE construct used in our study (62). These findings and our results indicate a low-dose effect, which is induced by low or moderate LET radiation, and may correspond to an early onset of tissue regeneration but does not occur at high doses and high LET, where cell cycle arrest and terminal differentiation are dominating.

### Obvious Changes in the Organization of the Basal Layer Occur After Exposure to Low Doses of X-Rays

In addition to changed differentiation and proliferation, we observed a radiation-induced transition from the typical palisade-like to a cobblestoned morphology of the basal cells for X-rays and carbon ions (Figure 5A). This is independent of the anchorage to the basal membrane, indicating a changed polarity of the basal cells. Semiquantitative analysis revealed a more pronounced effect for low compared to higher doses and comparing the same physical doses, a more pronounced effect for X-rays than for carbon ions (see Figure 5B) and comparing the same physical doses, a more pronounced effect for X-rays than for carbon ions.

A changed polarity has been characterized as a cellular change concomitant to the onset of proliferation and/or to migration (64), in particular in carcinogenic development. Anchorage-independent growth of epidermal cells can be evoked by irradiation as established in a murine epidermal cell line. Interestingly, we detected a delocalization of E-Cadherin from the cytoplasma membrane in HSE after X-ray and carbon ion exposure (Figure 5C). E-Cadherin is involved in cell–cell contacts of keratinocytes, and the transition to a cobblestoned morphology of the basal keratinocytes implies a dissociation of the intercellular contacts in the basal layer. The translocalization of E-Cadherin could be involved in the molecular mechanisms of radiation-induced anchorage independence, which was observed in our study. According to the results obtained so far, changed epidermal tissue organization plays a role for both X-ray and carbon ion exposure.

## Conclusion

Our results show that ionizing irradiation has an effect on the differentiation and organization of the epidermal layers in the skin equivalent. Densely ionizing charged particle are more effective than X-rays per unit dose in the induction of several biological endpoints, including DNA damage, chromosome aberrations, mutations, and cell killing. Our results suggest that exposure to carbon ions under therapy-like conditions triggers proinflammatory signals and changes in homeostasis and epidermal tissue organization to a similar extent as photons, independent of cell death. On the other hand, heavy ions and X-rays modify epidermal tissue organization at low doses and differentiation at high doses. How these tissue-specific effects can be related to the initial DNA damage, whose quality is different after low and high LET radiation, is unclear yet. Recently, Kang et al. (65) have shown that DNA damage response activates the GATA4 pathway, thus inducing inflammatory responses and reducing proliferation. The establishment of a direct link between DNA repair and late changes in homeostasis is important to explain why some effects can be differently revealed at low/high doses or low/high LET.

## Conflict of Interest Statement

The authors declare that the research was conducted in the absence of any commercial or financial relationships that could be construed as a potential conflict of interest.

The reviewers, Michael Wayne Epperly and Christopher James Bakkenist, and handling Editor Joel S. Greenberger declared their shared affiliation, and the handling Editor states that the process nevertheless met the standards of a fair and objective review.

## Abbreviations

cl, cleaved; D, dermis; fl, full length; HSE, human skin equivalent; M, marker; PC, positive control; SB, stratum basale; SC, stratum corneum; SG, stratum granulosum; SS, stratum spinosum.
