# Supplementary material for: Exposure to Carbon Ions Triggers Proinflammatory Signals and Changes in Homeostasis and Epidermal Tissue Organization to a Similar Extent as Photons
Source: Front Oncol. 2016 Jan 8;5:294. doi: 10.3389/fonc.2015.00294 (PMC4705223; doi:10.3389/fonc.2015.00294)
Supplement: Supplementary file 1 [file presentation_1.pdf]

Supplementary Material

Exposure to carbon ions triggers pro-inflammatory signals, changes in homeostasis and epidermal tissue organization to a similar extent as photons

Palma Simoniello, Julia Wiedemann, Joana Zink, Eva Thönnes, Maike Stange, Paul G. Layer, Maximilian Kovacs, Maurizio Podda, Marco Durante, and Claudia Fournier*

Correspondence: Claudia Fournier: c.fournier@gsi.de

1 Supplementary materials and methods

1.1 Cell culture

Normal Human Epidermal Keratinocytes (NHEK) purchased from Lonza (Walkersville, USA) were cultivated in KGM-gold (Lonza; Walkersville, USA) at standard conditions (37 °C, 5 % CO2 and 97 % humidity). Cells were sub-cultured after reaching a density of 70-80 % using a subculturing-kit (Lonza; Walkersville, USA) including trypsine, trypsine-neutralization-solution (TNS) and HEPES buffered saline solution (HBSS). One day before irradiation 350,000 cells were seeded in T25 flasks or 70,000 in CD35 dishes respectively.

Co-culture of NHEK and Normal Human Dermal Fibroblasts (NHDF) (Lonza; Walkersville USA) from the same donor was performed in 6 well plates with inserts (pore size 1 µm, BD Falcon, Heidelberg, Germany). About 100,000 NHDF were seeded in an insert (4.2 cm2) and cultivated in FGM Fibroblast Growth Media Kits FGM medium (FGM; Lonza, Walkersville, USA) for 4 days, until they reached confluence. About 75,000 keratinocytes per well (10 cm2) were seeded in 6-well plate with Keratinocyte Growth medium (KGM; Lonza, Walkersville, USA). Inserts with confluent NHDF were transferred in 6 well plates with keratinocytes and cultured with a 1:1 mixture of KGM and Eagel´s Minimal Essential Medium (EMEM; Lonza, Walkersville, USA) medium. The co-cultures were kept under standard conditions and were irradiated one day after assembly.

1.2 Normal skin organotypic culture

Human full-thickness skin equivalent constructs (EpiDermFTTM), referred to as HSE herein, were purchased from MatTek Corporation (Ashland, MA, USA). Details of culturing are described in the paper.

1.3 Irradiation

Cells were irradiated with X-rays, monoenergetic 170 keV/µm carbon ions or 120 keV/µm carbon ions (using a pencil beam in a spread out Bragg peak with 20.0 mm width equivalent to a depth of 5 cm in water; 120-135 MeV/u; rising flank of the Bragg Peak; 0,5 and 2 Gy).

At the time of exposure the cells were kept in medium Dulbecco's Modified Eagle Medium (DMEM; Biochrom, Berlin, Germany) supplemented with 1% penicillin/streptomycin (Biochrom; Berlin, Germany). Cells were typically analyzed 24, 48, 72 or 144 hours, in some experiments 1, 6 or 12 hours after irradiation.

UV-B irradiation of cells and HSE was performed using UV-B lamps with a peak at 312 nm and a range from 280-320 nm (Biolink-BLX-312, Vilber Lourmat, Marne-la-Vallée, France), operating with an intensity rate of 109 mJ/cm² per minute. In this spectrum 30 % of UV-A are included but this amount can be neglected due to the much higher MED for UV-A which is in the range of several J/cm². The culture medium of cells and HSE was replaced by PBS (Biochrom; Berlin, Germany) during the exposure to 15, 40, 60 or 100 mJ/cm2. PBS was replaced immediately after treatment by fresh, pre-warmed cell culture medium.

1.4 Clonogenic survival

Clonogenic survival after irradiation with X-rays or carbon ions (170 keV/µm) was determined as previously described (1).

1.5 DAPI and Annexin V/PI staining

NHEK were fixed (4 % paraformaldehyde) and stained with 4',6-diamidino-2-phenylindole (DAPI). Cell nuclei were examined microscopically and aberrant morphological features were classified, i.e. anaphase bridges, micronuclei (according to (2)), multinuclei and giant nuclei.

Apototic cells were detected using Annexin apoptosis detection kit (MACS Miltenyi Biotec; Bergisch Gladbach, Germany) as described by the manufacture's instruction. Apoptotic cells were identified by positive staining for Annexin V-FITC that binds to phosphotidylserine (PS). Necrosis or late stages of apoptosis were identified by positive Annexin V-FITC and PI staining, whereas viable cells were negative for both Annexin V-FITC and PI. About 600-700 cells for each dose and time point were analyzed.

1.6 Flow Cytometric anaysis of cell cycle distribution

To measure the cell cycle distribution after irradiation, NHEK cells were rapidly rinsed with PBS followed by trypsin dissociation (10 min at 37 °C). About 10,000 cells for each dose and time point were fixed with 1 ml ice-cold ethanol (70 %) and kept at -20 °C for 24 hours. After the fixation, the cells were centrifuged (200 g, 8 min., 4 °C), rinsed in PBS, centrifuged again and stained with 2 µg/ml of DAPI solution (AppliChem, Darmstadt, Gemany) for 30 min at room temperature in the dark. The fluorescence intensities of at least 10,000 cells per dose and time point were measured using the BD FACS Canto™ II Flow Cytometry System (BD; Heidelberg, Germany). Apoptotic cells were identified based on the subG1 DNA content. Data analysis was performed with the appropriate software package FlowJo (FlowJo, LLC; Ashland, OR, USA).

1.7 Western blot analysis and immunodetection

For the detection of expressed proteins, western blot analysis and immunodetection were carried out. Cells were prepared for western blot analysis at the indicated times after irradiation; the procedure was as previously described (3), except the lysis buffer, which was composed of 150 mM NaCl, 50 mM Tris pH 8.0, 1 % Triton X-100; protease inhibitor cocktail (Roche, Mannheim, Germany) was freshly added. Western blot and immunodetection were performed like described in the main part of the paper for HSE. The same antibodies were used.

1.8 Enzyme Linked Immunosorbant Assay (ELISA)

The supernatants of the cell cultures were centrifuged and medium was collected for ELISA-measurement. Trypsinized cells were counted with a Particle Count&Size Analyzer Coulter Z) (Beckman Coulter GmbH; Krefeld, Germany). ELISA was performed as described in the paper for the same cytokines. Cytokine release was calculated for 10,000 cells and normalized on the controls.

2 Supplementary results

Our study dedicated to investigate cell death, signals related to inflammation, and changes in homeostasis and epidermal tissue organization. UV-B exposure was used as a reference, because more studies are available for UV compared to ionizing irradiation. The UV-B intensities typically used were between 15 and 60 mJ/cm2, in some experiments additionally 100 mJ/cm2.

2.1 Cell death

In contrast to X-ray and carbon ion irradiation, apoptosis was clearly detected in NHEK cells by Annexin/PI staining at 24 hours and, to a small extent, at 144 hours after UV-B exposure (Figure S3A). Noteworthy, the induction of chromosomal damage in terms of micronuclei formation was less pronounced compared to X-ray and carbon ion exposure (Figure S1, S2), which points to a different regulation of apoptosis induction after UV-B exposure. The percentage of SubG1 cells was also increased 24 hours after UV-B exposure, peaking at 48 hours (Figure S3B). The elevated levels persisted up to the end of the observation period (144 hours). And finally, the early induction of apoptosis was confirmed in western blot analysis by the detection of activated (cleaved) caspase-3 and cleaved PARP 12 hours after exposure. The respective levels were lower at 24 hours and undetectable at 72 hours; for the highest UV-B intensity no proteins were detectable anymore (Figure S3C).

In more complex model systems of skin, i.e. the co-culture with NHDF (Figure S4), no indication of UVB induced apoptosis was found (western blot analysis of active (cleaved) caspase-3). This also applies for HSE, as can be inferred from representative images of HSE tissue sections (Figure S9, UV-B part of Figure 1A,B), stained at 24 hours after exposure to high doses UV-B intensities for in situ immuno-detection of activated, cleaved caspase-3.

Similarly, an induction of necrosis was found in NHEK cells, but not in co-cultures with NHDF or in HSE (not shown). Figure S5 shows that UV-B exposure of NHEK cells led to a clear release of HMGB1 (High Mobility Group Box 1 protein) 24 hours after exposure. This was not the case when NHEK were co-cultured with NHDF, pointing to a higher radio-resistance of the more complex

model system. This can also be inferred by a comparison of the intrinsic level of released HMGB, which was 5-fold higher in monolayer compared to co-cultures of HMGB.

2.2 Cytokine release

The cytokine release in NHEK monocultures after UV-B irradiation was not changed dramatically for IL-1α, IL-2 and TGF-β (Figure S7). Even if the reduction of IL-2 and TGF-β is significant after irradiation with 60 mJ/cm2 UV-B the reduction was only 0.8 fold compared to the control. The release of IL-6 and IL-8 was not detectable in the controls. But the IL-6 release was induced intensity-dependent whereas the release of Il-8 can be induced with an intensity of 60 mJ/cm2.

In co-cultures of NHEK and NHDF, a significant increase in the release of IL-1α, and a very pronounced increase of IL-6 and IL-8 were measured. The response was much more pronounced than for X-ray exposure, where also significant increments were detected (Figure S7), but the pattern was similar.

In contrast to the strong response of the co-culture, the UV-B induced effect observed in the 3D HSE, was moderate (Figure S10). After exposure, the levels of IL-1α were unchanged and a trend for an increased release of IL-6 and IL-8 was observed at 24 hours, while the levels of all cytokines dropped below the levels of the controls or were equivalent to controls at 48 hours after exposure.

2.3 Modified differentiation and organisation of the epidermal layer

The normal differentiation and migration process implies nuclear disintegration of the keratinocytes that have reached the stratum corneum (4). The differentiation process is considered to be abnormal if nucleated, pyknotic cells are found in the stratum corneum (parakeratosis) (Figure 3A). Likewise our observations for ionizing irradiation, we found that UV-B exposure induced a pronounced, albeit not significant increase in parakeratotic cells, which occurred later than for ionizing irradiation (72 hours, Figure S11A) Another aberrant feature of skin are so-called “sunburn cells” (shown in Figure 3C), which are not only characterized by pyknotic nuclei, but also by a eosinophil cytoplasm and, according to some studies, activated caspase-3 (5). Consistent with published results, cells with these characteristics were induced in a HSE by UV-B irradiation. Quantification, i.e. counting the number of sunburn cells in the layers of the viable epidermis per field of view, revealed that this occurred to a similar extent as for ionizing irradiation (Figure S11A). However, in contrast to (5), we could not detect activated, cleaved caspase-3 in these cells.

Furthermore, thickening of the stratum corneum was reported as a response to UV-B exposure (6). In Figure 3E, a thickened stratum corneum of an irradiated HSE compared to a control HSE is shown. For quantification, we measured the thickness of the stratum corneum and normalized this value to the thickness of the viable epidermis. In our experiments, only a trend for a thickened stratum corneum was visible (Figure11A), which is in contrast to our observations for ionizing irradiation.

Proliferation activity of the basal keratinocytes is part of the differentiation process. It was measured by Ki67 staining 72 hours after irradiation of HSE as depicted in Figure 4A. Quantification of the fraction of Ki67 positive cells and normalization on the level in non-irradiated HSE showed small and non-significant changes in the fraction of Ki67 positive cells following exposure to UV-B (Figure S11B, UV-B part of Figure 4B).

After exposure to ionizing irradiation we observed a transition from the typical palisade-like morphology of basal cells to a cobblestoned morphology (Figure 5A). Following UV-B exposure (Figure S11C) the described transition occurred as well, but with an effect increasing with the intensity of UV-B exposure.

We observed an additional change indicating a loss of polarity of basal keratinocytes, i.e. a delocalisation of E-Cadherin from the cytoplasmic membrane to the cytoplasm (Figure S11D) and first quantification results suggested a concomitant decreased overall expression of E-Cadherin (not shown) after UV-B irradiation.

3 Supplementary discussion

UV-B exposure of human skin was investigated for selected endpoints in model systems of different complexity, i.e. human keratinocytes (NHEK), a co-culture of NHEK with NHDF, and a human skin equivalent (HSE). The main goal of the study was to compare early inflammatory effects and impact on the homeastasis of two ionizing radiation qualities (X-ray, carbon ions) and UV-B was used as a reference irradiation. This implies that the UV-B induced changes observed herein are not intended to add substantial new information to published results.

Cell death plays a role in triggering the inflammatory response (7,8), and for UV exposure also inhibiting effects elicited in irradiated skin are known, involving resident immune cells and keratinocytes (9,10) and are suggested to have an impact on chronic inflammation (11). From our results obtained for ionizing irradiation we conclude that apoptosis and necrosis do not contribute to inflammatory reactions during the first days after exposure.

To validate these findings we also used UV-B exposure. In contrast to NHEK monolayer cultures, we could not detect apoptosis in co-cultures and HSE after UV-B irradiation. The results obtained in NHEK are in agreement with published data (12). With the exception of one study in epidermal skin equivalents (5), the published results on the occurrence of UV-B induced apoptosis are not opposed to our results, because either the UV-B intensities used were higher (13), or were combined with UV-A (14). In some studies caspase-3 was not investigated and apoptosis was inferred from TUNEL staining (15), which in our and independent hands (16) turned out to be inappropriate for the detection of apoptosis in keratinocytes. However, the variability and incompatibility of the radiation parameters and methods used shows that the use of UV-B as a reference irradiation was not suitable with respect to the validation of our results on ionizing radiation induced apoptosis.

We concluded from our study in the different model systems of skin that the release of the pro-inflammatory cytokines that could be detected after X-ray irradiation is not more enhanced after carbon ion exposure. Also for UV-B irradiation, for the co-culture of keratinocytes, a pronounced release of IL-1α, IL-6 and IL-8 was measured and is in agreement with results from other studies

(12). This accounts also for HSE; an enhanced release of IL-6 and IL-8 was measured (Figure S10), which is in accordance with published data (17,18).

Most of the observed changes in differentiation and organization of the basal layer after UV-B exposure are, as far as they have been investigated by others, comparable to the results from published studies. According to our own results, enhanced parakeratosis (13), and cells with pyknotic nuclei and a eosinophilic cytoplasm were reported for suprabasal parts of the viable epidermis (“sunburn cells”, (14,19,20)). However, we did not detect “sunburn cells” in the basal layer. In addition, these “sunburn cells” are often considered as apoptotic cells displaying caspase-3 activity (5,21). The discrepancy to our results with respect to the involvement of caspase-3 has already been discussed above.

Indication for non-aberrant, but accelerated differentiation after exposure to X-rays was obtained in our study. Quantitative analysis of thickness of the stratum corneum (hyperkeratosis) revealed only a trend for carbon ions and UV-B irradiation, indicating a dependence on radiation quality.

The observed transition from the typical palisade-like to a cobblestoned morphology, indicating changed polarity and anchorage-independent growth of the basal cells after exposure to ionizing radiation, was also detectable after UV-B exposure, but without a clear dependence on the intensity (Figure 5 D). However, the nuclear translocation of E-Cadherin that was also detected in UV-B exposed HSE may indicate that the changed organization of the basal cells plays also a role after UV-B exposure of skin.

4 References

1. Fournier C, Scholz M, Weyrather WK, Rodemann HP, Kraft G. Changes of fibrosis-related parameters after high- and low-LET irradiation of fibroblasts. Int J Radiat Biol (2001) 77:713–22. doi:10.1080/095530000110045025

2. Fenech M, Chang WP, Kirsch-Volders M, Holland N, Bonassi S, Zeiger E. HUMN project: detailed description of the scoring criteria for the cytokinesis-block micronucleus assay using isolated human lymphocyte cultures. Mutat Res (2003) 534:65–75. Available at: http://www.ncbi.nlm.nih.gov/pubmed/12504755

3. Fournier C, Wiese C, Taucher-Scholz G. Accumulation of the cell cycle regulators TP53 and CDKN1A (p21) in human fibroblasts after exposure to low- and high-LET radiation. Radiat Res (2004) 161:675–84. Available at: http://www.ncbi.nlm.nih.gov/pubmed/15161352

4. Kolarsick PAJ, Kolarsick MA, Goodwin C. Anatomy and physiology of the skin. J Dermatology Nurses´Association (2011) 3:203–213.

5. Qin J-Z, Chaturvedi V, Denning MF, Bacon P, Panella J, Choubey D, Nickoloff BJ. Regulation of apoptosis by p53 in UV-irradiated human epidermis, psoriatic plaques and senescent keratinocytes. Oncogene (2002) 21:2991–3002. doi:10.1038/sj.onc.1205404

6. Elias PM. Stratum corneum defensive functions: an integrated view. J Invest Dermatol (2005) 125:183–200. doi:10.1111/j.0022-202X.2005.23668.x

7. Fink SL, Cookson BT. Apoptosis, pyroptosis, and necrosis: mechanistic description of dead and dying eukaryotic cells. Infect Immun (2005) 73:1907–16. doi:10.1128/IAI.73.4.1907-1916.2005

8. Rödel F, Frey B, Manda K, Hildebrandt G, Hehlgans S, Keilholz L, Seegenschmiedt MH, Gaipl US, Rödel C. Immunomodulatory properties and molecular effects in inflammatory diseases of low-dose x-irradiation. Front Oncol (2012) 2:120. doi:10.3389/fonc.2012.00120

9. Hart PH, Grimbaldeston MA, Finlay-Jones JJ. Sunlight, immunosuppression and skin cancer: role of histamine and mast cells. Clin Exp Pharmacol Physiol (2001) 28:1–8. Available at: http://www.ncbi.nlm.nih.gov/pubmed/11153522

10. Jans J, Garinis GA, Schul W, van Oudenaren A, Moorhouse M, Smid M, Sert Y-G, van der Velde A, Rijksen Y, de Gruijl FR, et al. Differential role of basal keratinocytes in UV-induced immunosuppression and skin cancer. Mol Cell Biol (2006) 26:8515–26. doi:10.1128/MCB.00807-06

11. Voll RE, Herrmann M, Roth EA, Stach C, Kalden JR, Girkontaite I. Immunosuppressive effects of apoptotic cells. Nature (1997) 390:350–1. doi:10.1038/37022

12. Petit-Frere C, Capulas E, Lyon DA, Norbury CJ, Lowe JE, Clingen PH, Riballo E, Green MHL, Arlett CF. Apoptosis and cytokine release induced by ionizing or ultraviolet B radiation in primary and immortalized human keratinocytes. Carcinogenesis (2000) 21:1087–1095. doi:10.1093/carcin/21.6.1087

13. Bernerd F, Asselineau D. Successive alteration and recovery of epidermal differentiation and morphogenesis after specific UVB-damages in skin reconstructed in vitro. Dev Biol (1997) 183:123–38. doi:10.1006/dbio.1996.8465

14. Kandarova H, Armento A, Stolper G, Cooney C, Li M, Hayden PJ. PARP-1 activity is involved in the solar UV-induced cutaneois inflammatory response in the EpiDerm-FT in vitro human skin model. SOT (2011) Abstract #:1–4.

15. Su Y, Meador JA, Geard CR, Balajee AS. Analysis of ionizing radiation-induced DNA damage and repair in three-dimensional human skin model system. Exp Dermatol (2010) 19:e16–22. doi:10.1111/j.1600-0625.2009.00945.x

16. Ahmed EA, Agay D, Schrock G, Drouet M, Meineke V, Scherthan H. Persistent DNA damage after high dose in vivo gamma exposure of minipig skin. PLoS One (2012) 7:e39521. doi:10.1371/journal.pone.0039521

17. Varnum SM, Springer DL, Chaffee ME, Lien KA, Webb-Robertson B-JM, Waters KM, Sacksteder CA. The effects of low-dose irradiation on inflammatory response proteins in a 3D reconstituted human skin tissue model. Radiat Res (2012) 178:591–9. doi:10.1667/RR2976.1

18. Breger J, Baeva L, Agrawal A, Shindell E, Godar DE. UVB-induced inflammatory cytokine release, DNA damage and apoptosis of human oral compared with skin tissue equivalents. Photochem Photobiol 89:665–70. doi:10.1111/php.12030

19. Lippens S, Hoste E, Vandenabeele P, Agostinis P, Declercq W. Cell death in the skin. Apoptosis (2009) 14:549–69. doi:10.1007/s10495-009-0324-z

20. Lin F-H, Lin J-Y, Gupta RD, Tournas JA, Burch JA, Selim MA, Monteiro-Riviere NA, Grichnik JM, Zielinski J, Pinnell SR. Ferulic acid stabilizes a solution of vitamins C and E and doubles its photoprotection of skin. J Invest Dermatol (2005) 125:826–32. doi:10.1111/j.0022-202X.2005.23768.x

21. Van Laethem A, Claerhout S, Garmyn M, Agostinis P. The sunburn cell: regulation of death and survival of the keratinocyte. Int J Biochem Cell Biol (2005) 37:1547–53. doi:10.1016/j.biocel.2005.02.015

5 Supplementary Figures and Legends

Figure S1: Detection of clonogenic survival, apoptosis and micronuclei in NHEK 24, 72 and 144 hours after irradiation with X-ray **(A):** Clonogenic survival of NHEK after X-ray and carbon ion (170 keV/µm) irradiation **(B):** NHEK stained with DAPI (micronuclei) and Annexin/Propidium iodite; dose-dependent formation of micronuclei is observed; almost no annexin and/or PI positive cells indicating primary apoptosis and/or necrosis were found; DAPI staining: N=2, n=4 ; Annexin/PI: n=1 (500-600 cells); **(C):** No elevated percentage of cells with Sub-G1 DNA content; N=3, n=4; 10,000 cells/data point; error bars: standard deviation **(D):** Western blot analysis of caspase-3 and PARP; no cleaved caspase-3 and PARP were detected in NHEK after irradiation; N=2, n=4

Figure S2: Detection of apoptosis and micronuclei in NHEK cells 12, 24, 72 and 144 hours after irradiation with carbon ions (A) NHEK stained with DAPI (micronuclei) and Annexin/Propidium iodite**;** dose-dependent formation of micronuclei but no clear enhancement of Annexin or PI positive cells, indicating primary apoptosis and/or necrosis were found; DAPI staining: N=2; n=4; Annexin/PI: n=1 (500-600cells); error bars: standard deviation **(B)** No elevated percentage of cells with Sub-G1 DNA content; N=3, n=4; 10,000 cells/data point; error bars: standard deviation **(C):** Western blot analysis of caspase-3 and PARP; no cleaved caspase-3 and PARP were detected in NHEK after irradiation; N=4 n=8

Figure S3: Detection of apoptosis and micronuclei in NHEK cells 12, 24, 72 and 144 hours after irradiation with UV-B (A): NHEK stained with DAPI (micronuclei) and Annexin/Propidium iodite, no micronuclei were found, elevated percentage of Annexin positive cells, indicating primary apoptosis were detected for 40 mJ/cm² and 60 mJ/cm² after 24 hours, DAPI staining: N=2; n=4; Annexin/PI: n=1 (500-600cells); error bars standard deviation **(B):** An increase of cells with Sub-G1 DNA content, indicating apoptotis, was found at 40 mJ/cm² and 60 mJ/cm², N=1, n=2; 10,000 cells/data point, error bars: standard deviation **(C):** Western blot analysis of caspase-3 and PARP.; active/cleaved caspase-3 and PARP are detected for 40 mJ/cm² and 60 mJ/cm² after 12 and 24 hours (circles); N=3, n=6

Figure S4: Western blot analysis of caspase-3 in co-cultures (NHEK and NHDF) 24 hours after irradiation with X-ray and UV-B Cleaved caspase-3 is not present; N=2, n=3

Figure S5: Detection of HMGB1 release from mono- and co-cultures 24 hours after irradiation with X-ray, C-ions and UV-B (A): HMGB1 release of monocultures normalized on controls shows a significant enhancement for 40 mJ/cm² **(B):** HMGB1 release in mono- compared to co-cultures revealed a 10-fold higher release in monocultures **(C):** HMGB release of co-cultures shows only slight radiation induced changes; N=2, n=2

Figure S6: Detection of apoptosis in situ and in western blot in HSE 6 hours to 72 hours after irradiation with X-ray and C-ions (A): Immunodetection of active caspase-3 (pink) in HSE 72 hours after X-ray and carbon ions; cleaved caspase-3 was only detected in the positive control (arrows point to the pink staining) **(B):** Western blot analysis of caspase-3 and PARP in HSE; apoptosis is detected by the presence of caspase-3 and PARP cleavage fragments (17 and 19 kDa; 89 kDa) in PCs; active/cleaved caspase-3 and cleaved PARP were not detected in the epidermis of HSE after irradiation; [1] and [2] represents two sample from the same experiment; N=3, n=5

Figure S7: Detection of cytokine release in mono- and co-cultures 24 hours after irradiation with X-ray and UV-B (A): Enhanced release of IL-2 and TGF-β in monocultures; IL-6 and IL-8 are not detectable in control cells, but are released after X-ray **(**IL-6) and UV-B exposure (IL-6 and IL-8), IL-1α is unchanged **(B):** Release of IL-1α and TGF-β in non-irradiated mono- and co-cultures shows an inhibitory feed-back loop between these cytokines **(C):** Cytokine release of co-cultures; radiation induced moderate increase in the release of IL-1α and an increment of the IL-6 and IL-8 after X-ray and UV-B is shown; $p \leq 0,05$= *, $p \leq 0,01$=**; $p \leq 0,001$=***; N=2, n=4

Figure S8: Cell cycle measurement of NHEK 6 to 144 hours after irradiation with X-ray and carbon ions (A and B): An accumulation of G2 phase cells is observed for high doses of both radiation qualities **(A):** N=2, n=4 **(B):** N=2, n=2; for 6h, 48h and 144 h n=1; SEM

Figure S9: Detection of apoptosis in situ and in western blot in HSE 24 hours an 72 hours after irradiation with UV-B (A): Immunodetection of active caspase-3 (pink) in HSE; cleaved caspase-3 was not detected in the epidermis after irradiation, but in the PCs (arrows) **(B):** Western blot analysis of caspase-3 and PARP in HSE; apoptosis is detected by the presence of caspase-3 and PARP cleavage fragments (17 and 19 kDa; 89 kDa) in PCs; active/cleaved caspase-3 and cleaved PARP were not detected in the epidermis of HSE after irradiation; N=3, n=5

Figure S10: D**etection of cytokine release by ELISA in HSE after irradiation with UV-B** Cytokine detection shows a fast irradiation effect with a slight enhancement of IL-6 and IL-8 24 hours after irradiation, whereas after 48 hours a slight decrease of IL-1α, IL-10 and TGF-β was detected; SEM; $p \leq 0,05$= *, $p \leq 0,01$=**; N=2-3, n=3-10; IL-2 and TNF-α were not detectable

Figure S11: Abnormal and accelerated differentiation, proliferation and changed polarity of basal cells after irradiation with UV-B (A): Quantification of parakeratosis, sun burned cells and hyperkeratosis in HSE 24 and 72 hours after UV-B irradiation **(B):** Quantification of Ki67 positive cells in the HSE **(C):** Quantification of palisade-like morphology and cobblestoned (partial or total) morphology shows a slight transition for all intensities **(D):** Immunolocalization of E-Cadherin

shows a delocalization in the basal cells (arrows) and a lower expression in the suprabasal layers 72 hours after UV-B irradiation; N=2, n=4
