# Supplementary figures and images for: Exposure to Carbon Ions Triggers Proinflammatory Signals and Changes in Homeostasis and Epidermal Tissue Organization to a Similar Extent as Photons
Source: Front Oncol. 2016 Jan 8;5:294. doi: 10.3389/fonc.2015.00294 (PMC4705223; doi:10.3389/fonc.2015.00294)

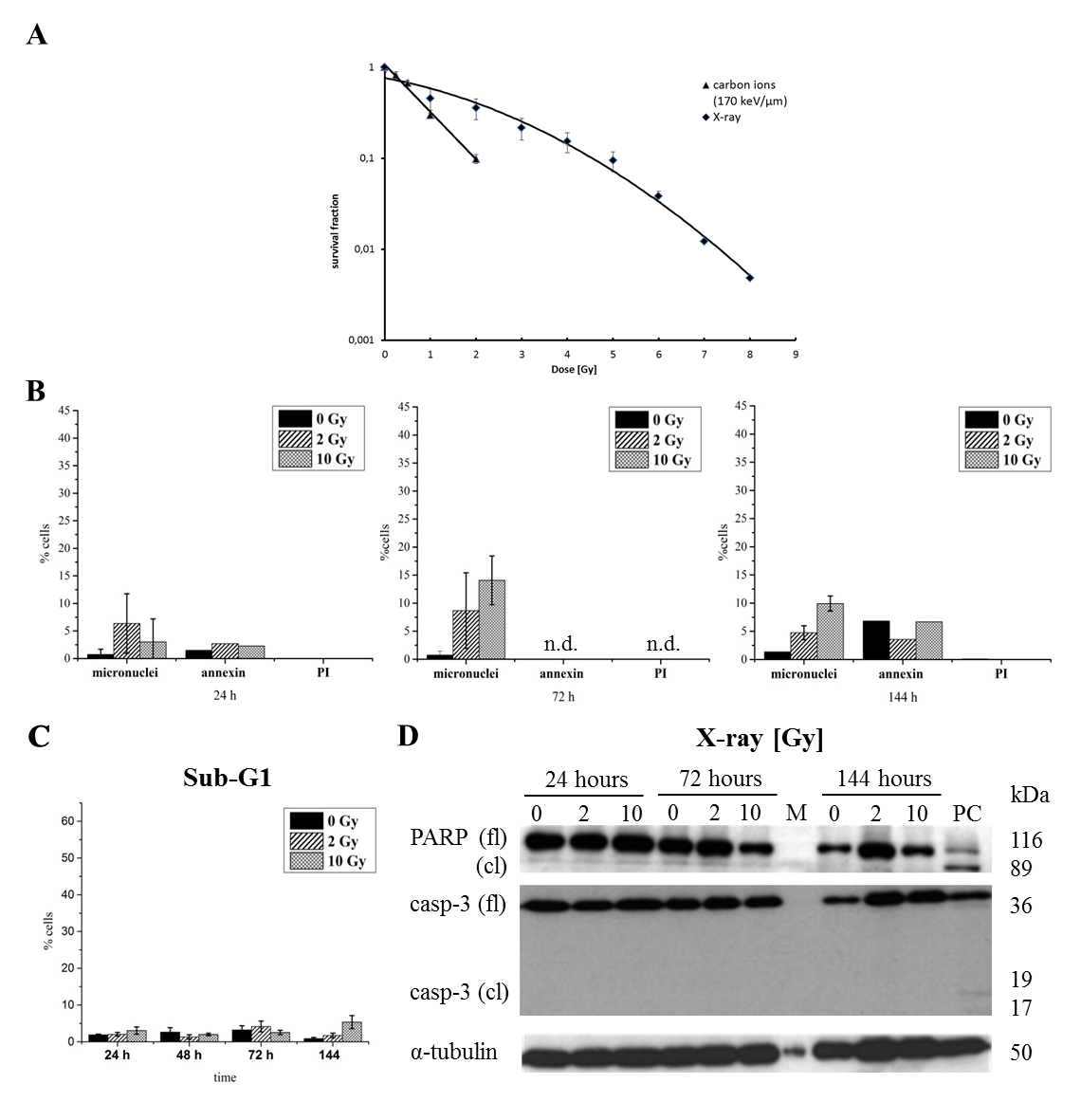

Supplement: Supplementary file 2 [file image_1.tif]

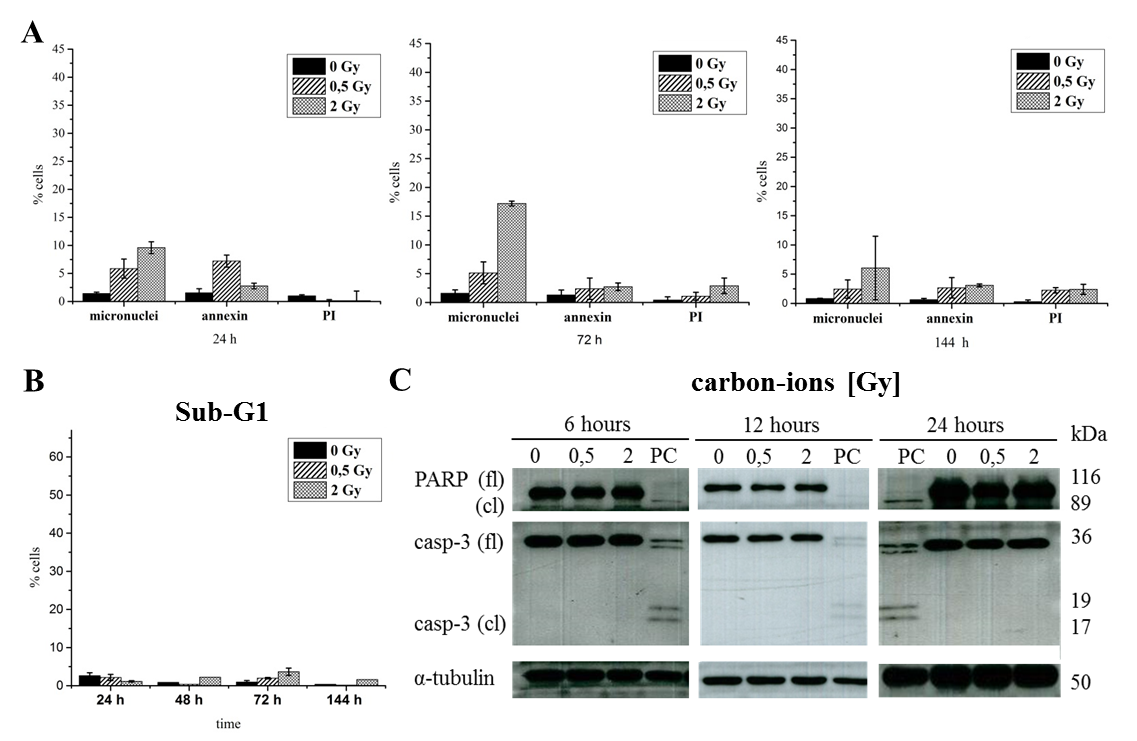

Supplement: Supplementary file 3 [file image_2.tif]

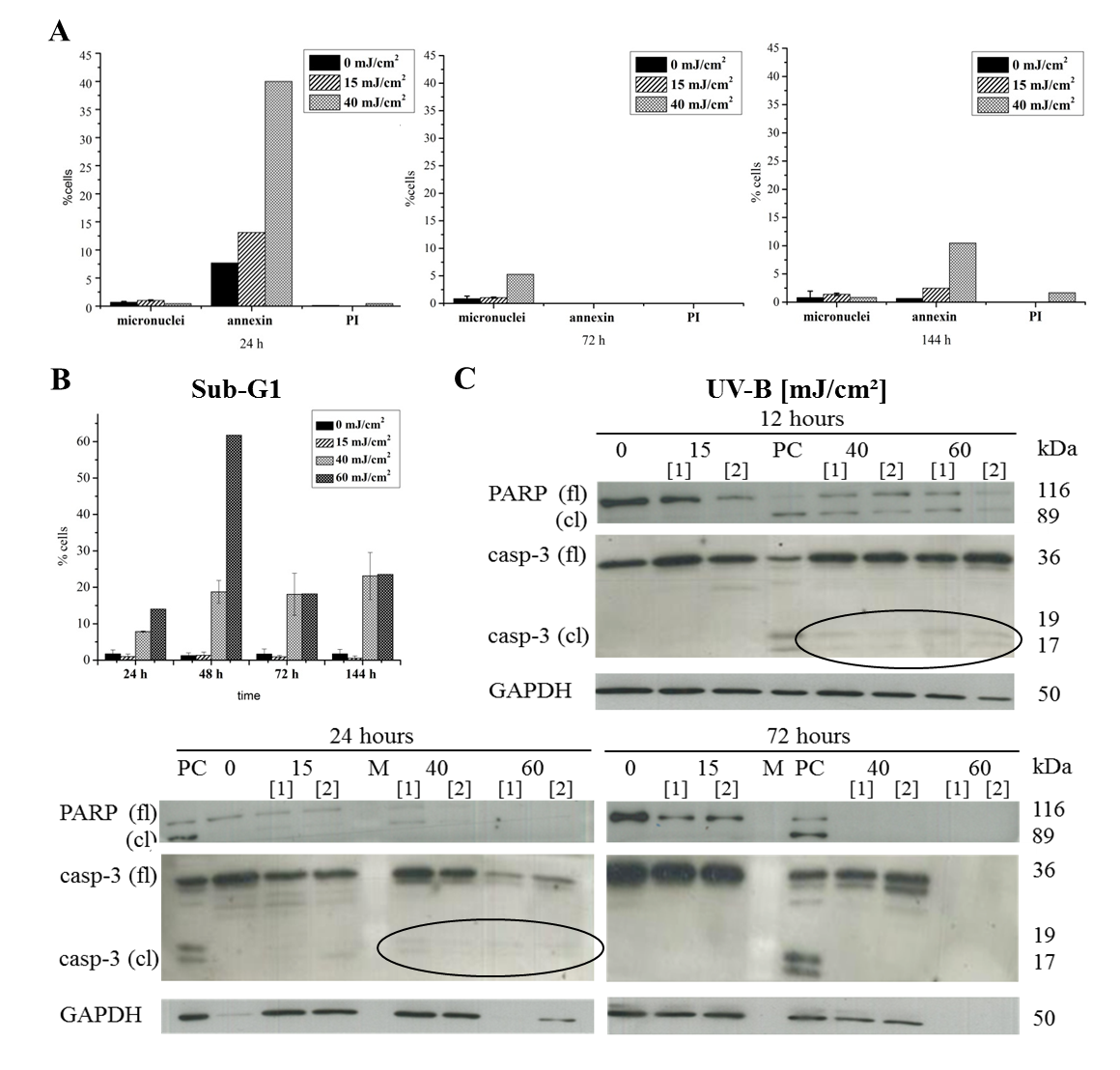

Supplement: Supplementary file 4 [file image_3.tif]

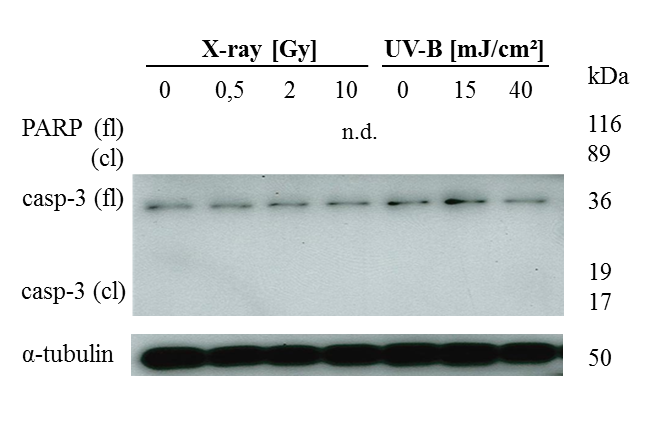

Supplement: Supplementary file 5 [file image_4.tif]

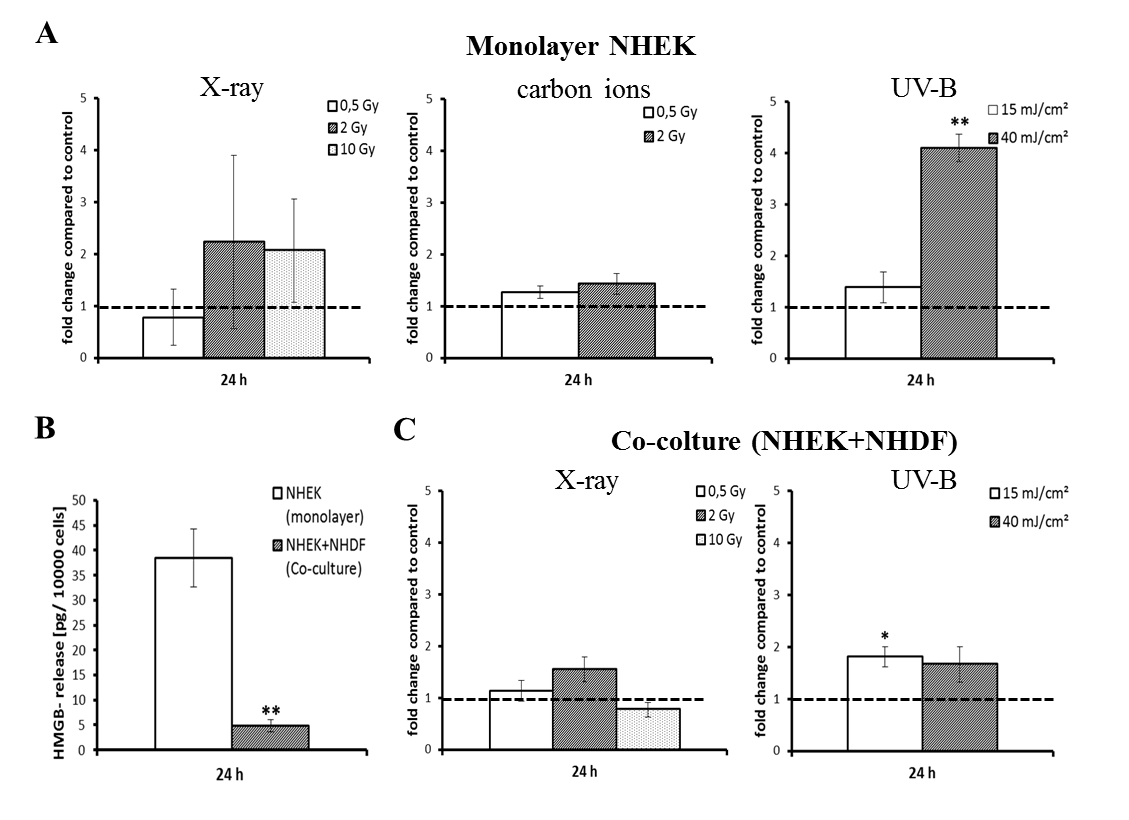

Supplement: Supplementary file 6 [file image_5.tif]

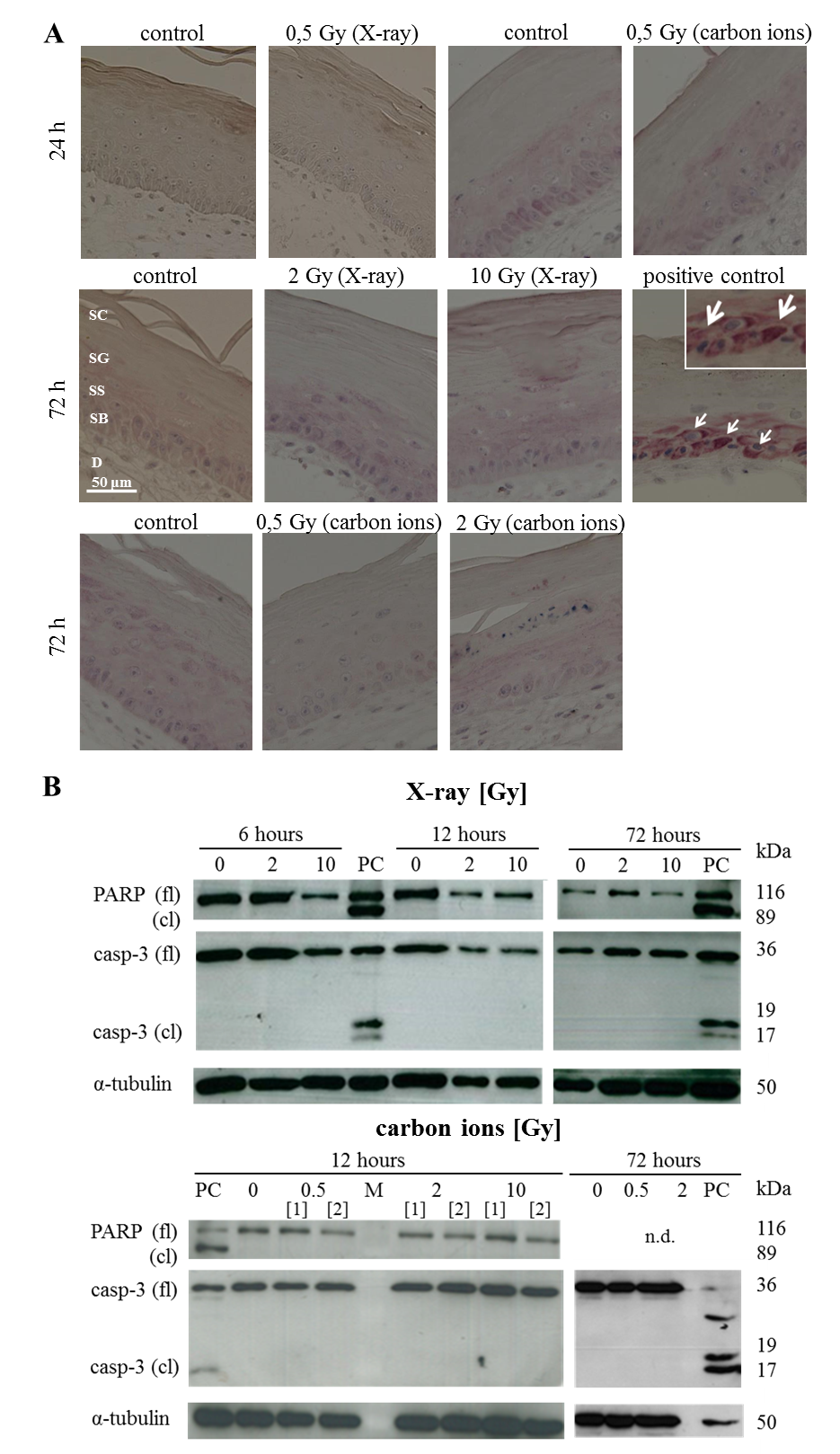

Supplement: Supplementary file 7 [file image_6.tif]

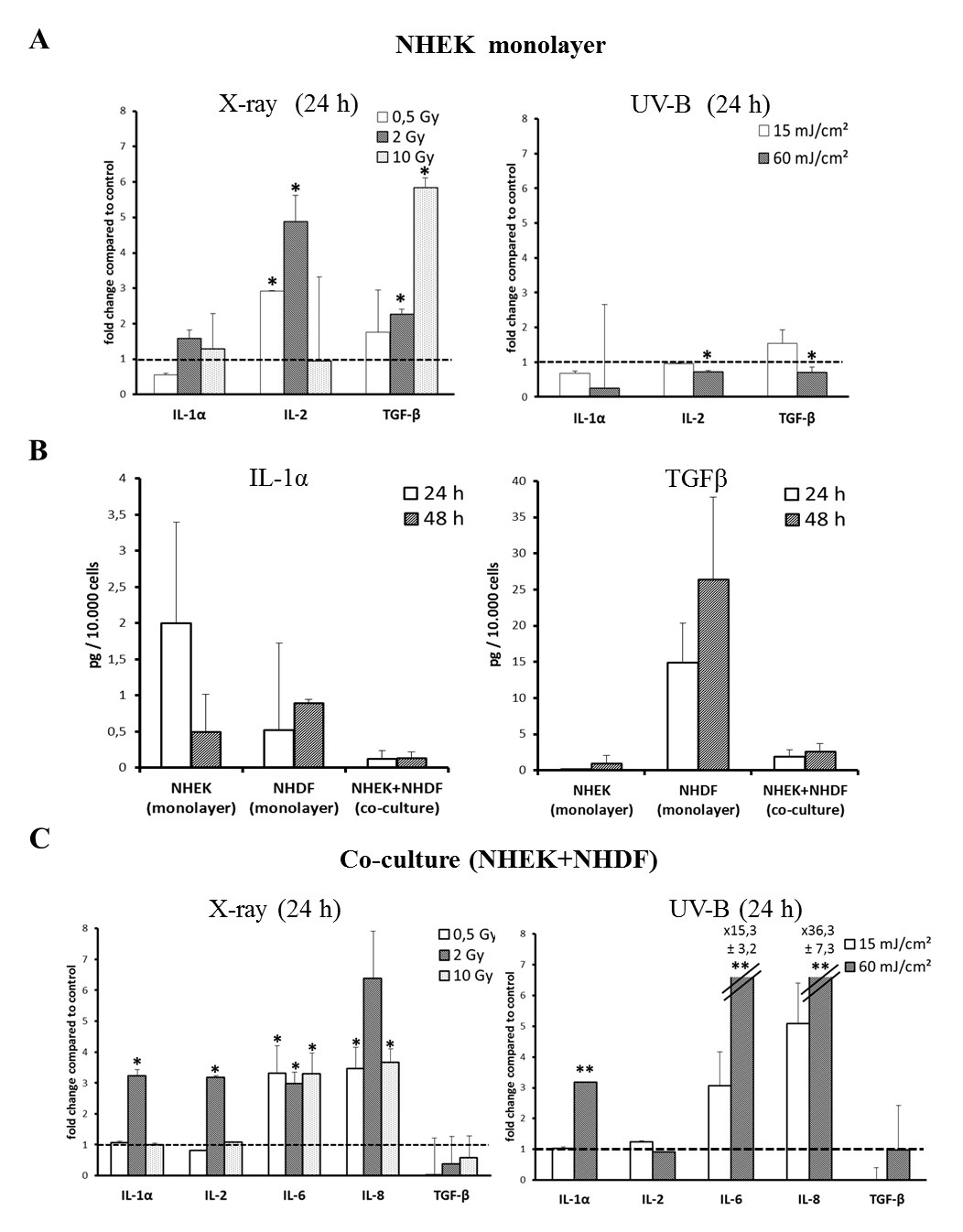

Supplement: Supplementary file 8 [file image_7.tif]

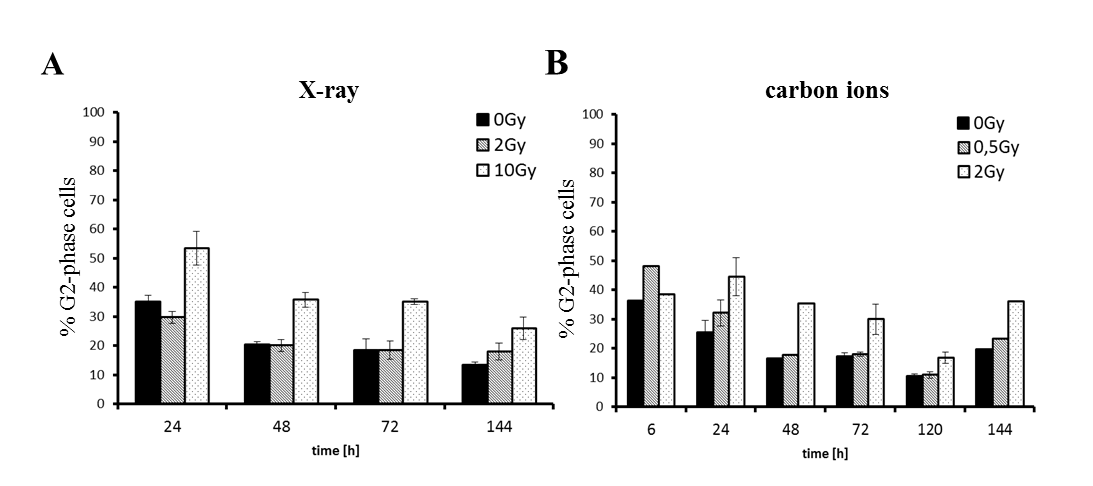

Supplement: Supplementary file 9 [file image_8.tif]

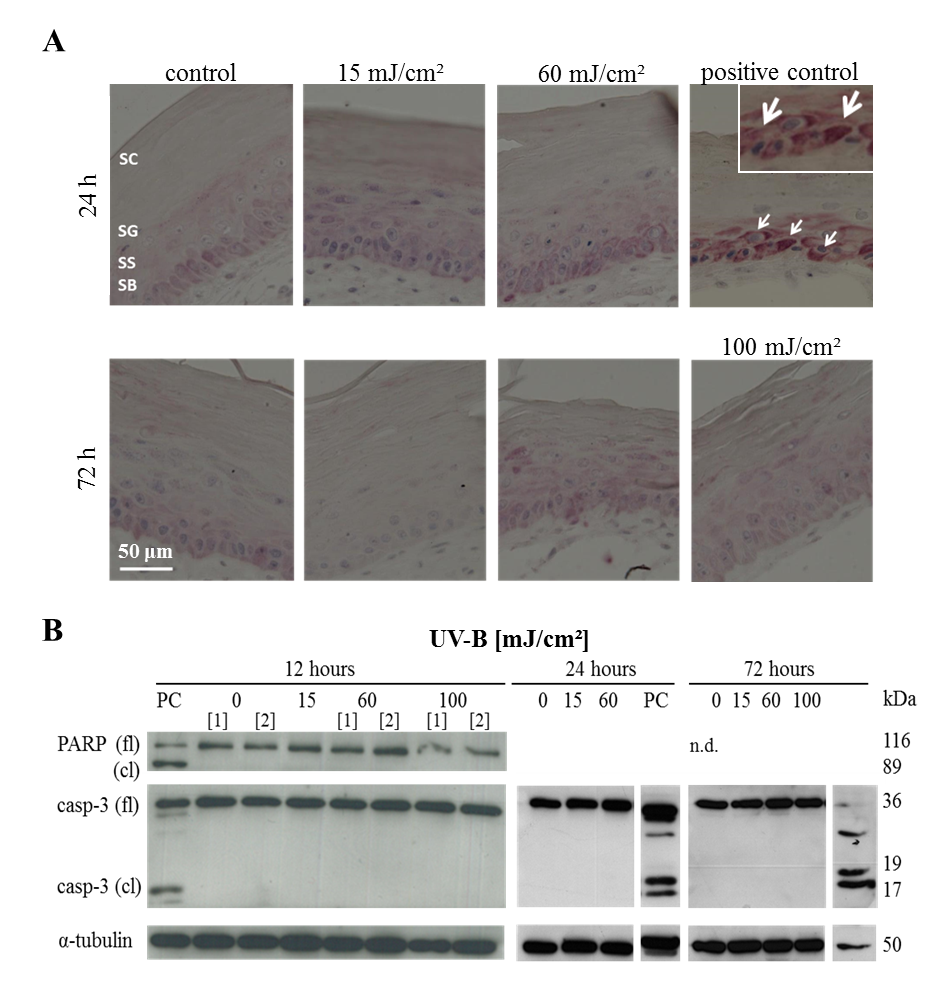

Supplement: Supplementary file 10 [file image_9.tif]

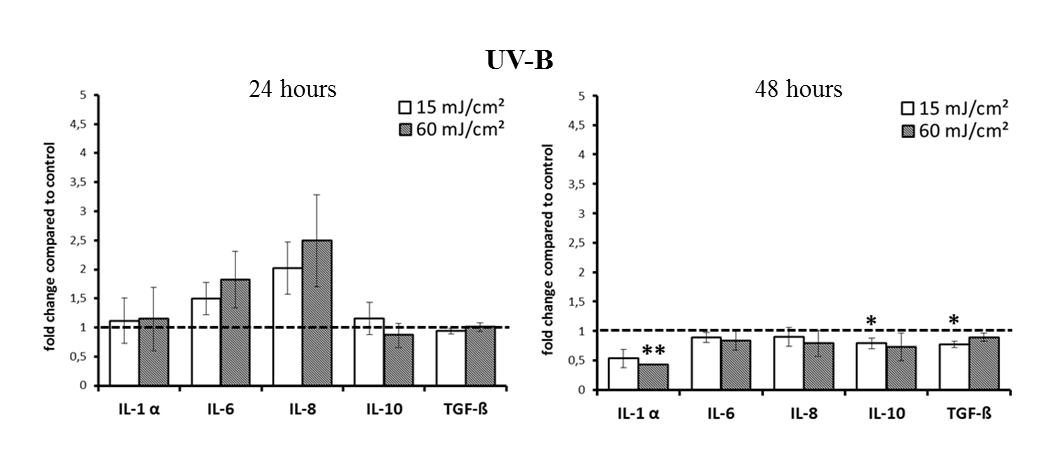

Supplement: Supplementary file 11 [file image_10.tif]

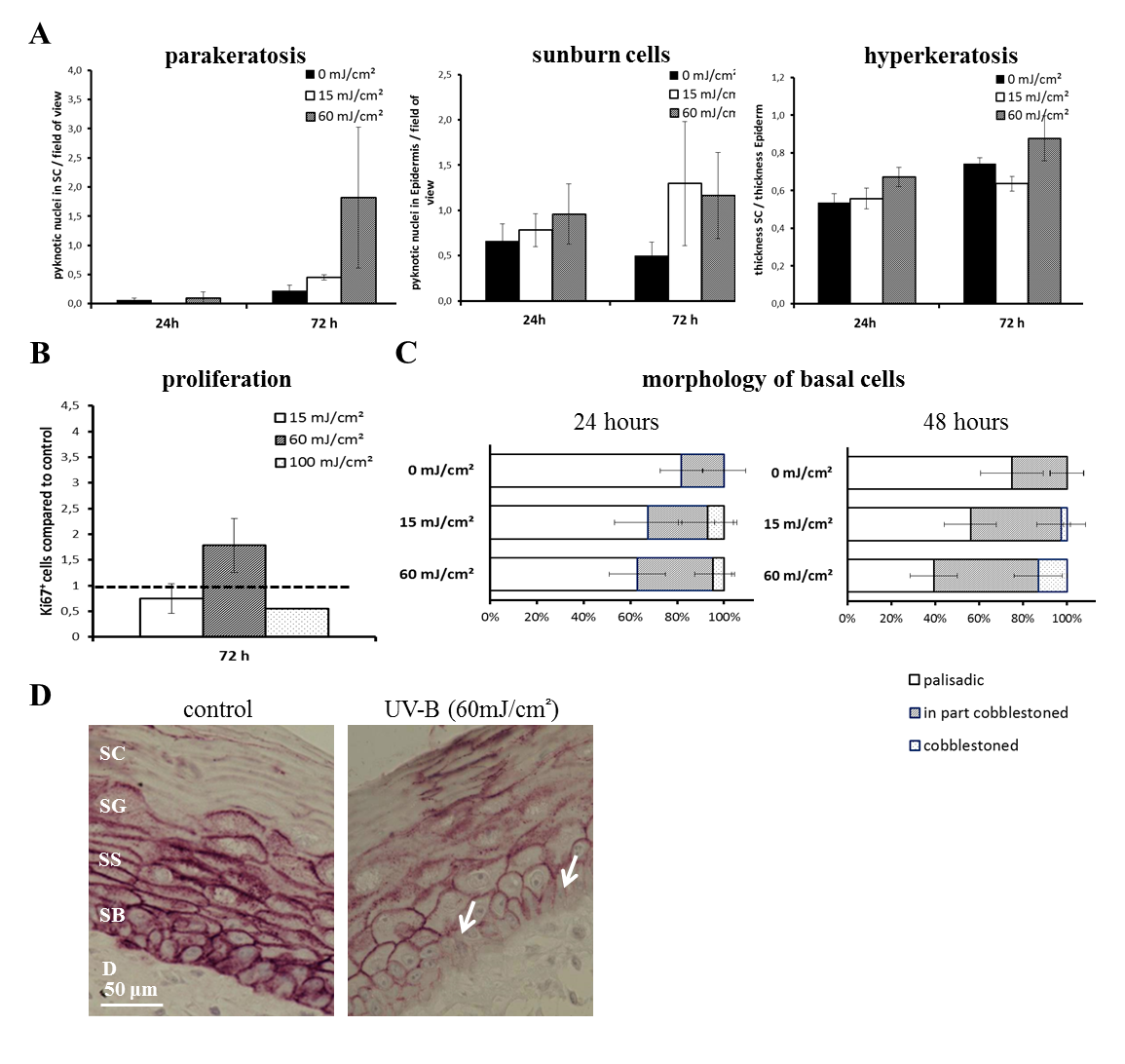

Supplement: Supplementary file 12 [file image_11.tif]
